# Patient-Controlled Real-Time Transcutaneous Electrical Acupoint Stimulation at Neiguan (PC6) for Chemotherapy-Induced Nausea and Vomiting in Breast Cancer: An Exploratory Single-Arm Two-Stage Trial

**DOI:** 10.3390/curroncol33090520

**Published:** 2026-08-31

**Authors:** Huiting Liu, Jie Zhou, Junying Du, Qiongying Shen, Chao Lu, Jianxing Zhang, Weiping Zhang, Ran Ran, Jianqiao Fang, Jiongyao Ye, Hong Gao, Junfan Fang

**Affiliations:** 1 The Third Clinical Medical College, Zhejiang Chinese Medical University, Hangzhou 310053, China; 202411111211029@zcmu.edu.cn (H.L.); 20181093@zcmu.edu.cn (J.D.); sqy@alu.zcmu.edu.cn (Q.S.); 2 Department of Acupuncture and Moxibustion, The Third Affiliated Hospital of Zhejiang Chinese Medical University, Hangzhou 310053, China; 20115021@zcmu.edu.cn; 3 Key Laboratory of Acupuncture and Neurology of Zhejiang Province, Department of Neurobiology and Acupuncture Research, The Third Affiliated Hospital of Zhejiang Chinese Medical University, Zhejiang Chinese Medical University, Hangzhou 310053, China; 19831012@zcmu.edu.cn; 4 Department of Traditional Chinese Medicine, Zhejiang Cancer Hospital, Hangzhou 310022, China; luchao@zjcc.org.cn; 5 School of Information Science and Engineering, East China University of Science and Technology, Shanghai 200237, China; y18232001@mail.ecust.edu.cn (J.Z.); yejy@ecust.edu.cn (J.Y.); 6 Department of Cancer, The Third Affiliated Hospital of Zhejiang Chinese Medical University, Hangzhou 310013, China; 19935007@zcmu.edu.cn (W.Z.); 20105018@zcmu.edu.cn (R.R.)

**Keywords:** breast cancer, chemotherapy-induced nausea and vomiting, transcutaneous electrical acupoint stimulation

## Abstract

Chemotherapy-induced nausea and vomiting (CINV) remain difficult to control after discharge despite guideline-based antiemetics. This multicenter exploratory single-arm two-stage trial evaluated patient-controlled, real-time transcutaneous electrical acupoint stimulation (TEAS) at Neiguan (PC6) as an adjunct to standard antiemetic therapy in patients with breast cancer. Forty-eight patients who had experienced CINV and were scheduled at enrollment to receive the same chemotherapy and antiemetic regimens during two consecutive cycles were analyzed. Cycle 1 served as the observation period, and cycle 2 added on-demand wrist-worn TEAS initiated by patients at nausea or vomiting onset during 0–120 h after chemotherapy. Compared with the observation period, TEAS was associated with lower nausea frequency and visual analogue scale scores on days 1–5 and higher nausea response rates on days 1–4. Vomiting frequency and severity were lower on days 1–3, but between-period differences were not significant on days 4–5. SF-36 scores increased and SAS/SDS scores decreased on day 5. Two transient local numbness events occurred, with no serious TEAS-related adverse events. These findings suggest that patient-controlled TEAS is feasible and warrants confirmation in randomized sham-controlled trials.

## Simple Summary

Chemotherapy-induced nausea and vomiting (CINV) remain common and distressing adverse effects of chemotherapy, even when guideline-recommended antiemetic medications are used. This study evaluated a wearable wrist-type transcutaneous electrical acupoint stimulation (TEAS) device that patients could activate themselves when symptoms occurred. In 48 patients with breast cancer receiving chemotherapy, patient-controlled TEAS was associated with lower nausea frequency and severity, improved nausea response rates, and modest improvements in quality-of-life measures. Differences in vomiting outcomes were mainly observed during the first three days after chemotherapy. The intervention was well tolerated, with only two transient cases of local numbness. These findings suggest that wearable, patient-controlled TEAS may represent a practical supportive-care strategy for outpatient management of chemotherapy-induced nausea and warrant further evaluation in randomized controlled trials.

## 1. Introduction

Chemotherapy-induced nausea and vomiting (CINV) is among the most common and distressing adverse effects of anticancer treatment. The emetogenicity of the chemotherapy regimen is a major determinant of CINV risk [1]. Many chemotherapy regimens used for breast cancer are classified as moderately or highly emetogenic, and reported CINV rates associated with these regimens can reach 70–90% [2]. Inadequately controlled CINV may lead to dehydration, electrolyte imbalance, malnutrition, reduced quality of life, and poor adherence to chemotherapy [3,4]. Although guideline-recommended antiemetic regimens have improved CINV control, 20–55.2% of patients continue to report inadequate relief [5,6]. In addition, antiemetic medications may be associated with adverse effects such as headache, constipation, and hepatic dysfunction [7]. Safe, convenient, and effective adjunctive strategies are therefore needed, particularly for symptoms that occur outside the hospital.

Acupuncture-point stimulation has been investigated as an adjunct to pharmacological antiemetic therapy [8,9]. International integrative oncology guidelines indicate that acupressure may be considered for chemotherapy-related vomiting, while acknowledging limitations in the available evidence [10,11]. International randomized trials of electroacupuncture, P6 acupressure, and electrical acustimulation wristbands have reported heterogeneous findings. Some studies have shown improvements in acute or delayed nausea or vomiting, whereas others have found no clear advantage over sham stimulation or standard care [12,13,14]. These inconsistent findings may reflect differences in patient populations, chemotherapy and antiemetic regimens, stimulation modalities, treatment schedules, and control-group designs.

Transcutaneous electrical acupoint stimulation (TEAS) is a non-invasive technique that delivers electrical stimulation through surface electrodes placed over selected acupoints. Electrical stimulation wristbands evaluated in previous international studies provide a relevant methodological precedent for wearable TEAS because both use non-invasive electrical stimulation at or near the wrist; however, the devices, stimulation parameters, treatment duration, and activation strategies are not necessarily equivalent [15,16,17]. Clinical studies of TEAS have suggested potential benefits for CINV [18], but the evidence remains limited by variations in intervention protocols and study design. Neiguan (PC6), located on the palmar aspect of the forearm, is one of the most commonly used acupoints for the management of nausea and vomiting [19,20]. From a biomedical perspective, stimulation at PC6 may influence autonomic function and gastric myoelectrical activity, although the mechanisms underlying its antiemetic effects remain incompletely understood [21,22]. From the perspective of traditional Chinese medicine, PC6 stimulation is used to regulate qi, harmonize the stomach, and relieve nausea.

Most previous studies of acupuncture-point stimulation for CINV have used prophylactic or predefined treatment schedules administered at fixed time points before or around chemotherapy [12,13,14,16]. This approach may not fully reflect real-world CINV patterns. CINV symptoms are often episodic, fluctuate across the 0–120 h post-chemotherapy period, and may occur after patients have left the hospital. With shorter chemotherapy-related hospital stays and increasing outpatient management in breast cancer care [23], patients may have limited access to timely nonpharmacological intervention when symptoms begin. Furthermore, fixed treatment schedules may not account for interindividual differences in symptom timing and severity. Evidence concerning electrical stimulation initiated by patients at the actual onset of nausea or vomiting remains scarce.

Patient-controlled, real-time TEAS may address these limitations by allowing patients to initiate stimulation when nausea or vomiting occurs. This approach may improve the timeliness of symptom relief, reduce reliance on in-hospital administration, and better match treatment intensity to individual symptom burden. To support this model, our team developed a portable wrist-worn TEAS device designed to deliver non-invasive PC6 stimulation with minimal disruption to daily activities.

Because evidence for symptom-triggered, patient-initiated TEAS in CINV remains limited, we conducted an exploratory multicenter, single-arm, two-stage clinical trial. Using a self-controlled design across two consecutive chemotherapy cycles, this study evaluated the feasibility, safety, and preliminary efficacy of real-time TEAS as an adjunct to standard antiemetic therapy in patients with breast cancer.

## 2. Materials and Methods

### 2.1. Study Design and Recruitment

**Figure 1:**
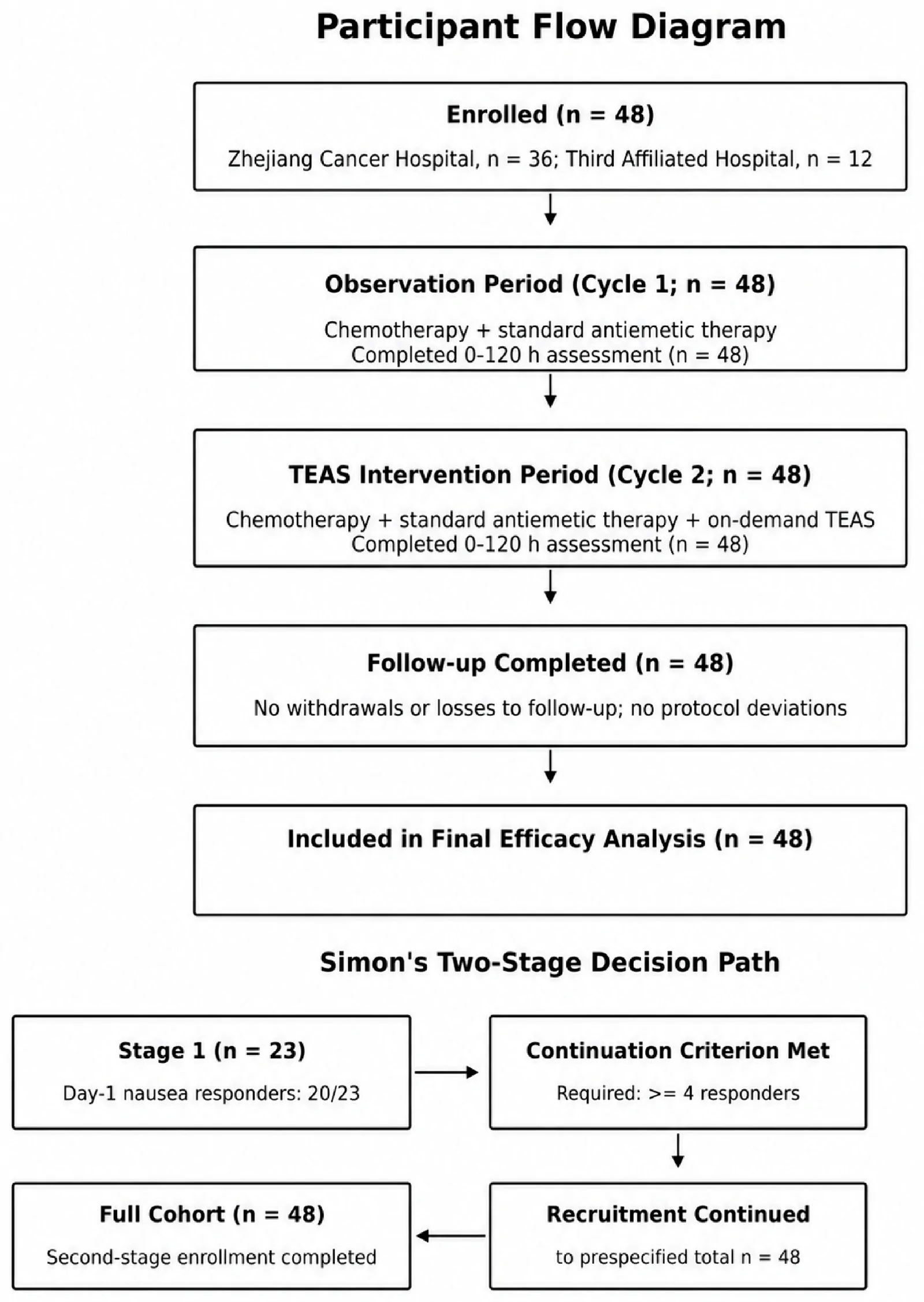
Participant flow diagram. A total of 48 participants were enrolled and completed the observation period (Cycle 1), the TEAS intervention period (Cycle 2), and follow-up, with no withdrawals or losses to follow-up. All 48 participants were included in the final efficacy analysis. In the first stage of Simon’s two-stage design, 20 of 23 participants met the prespecified day-1 nausea-response criterion, exceeding the continuation threshold and allowing recruitment to proceed to the full cohort of 48 participants. TEAS, transcutaneous electrical acupoint stimulation.

This was a multicenter, exploratory, single-arm, two-stage clinical trial evaluating the feasibility, safety, and preliminary efficacy of patient-controlled, real-time TEAS as an adjunct to standard antiemetic therapy for CINV. Participants were recruited from Zhejiang Cancer Hospital and The Third Affiliated Hospital of Zhejiang Chinese Medical University. Eligible patients entered two consecutive chemotherapy cycles. The first cycle served as the observation period, during which patients received chemotherapy and standard antiemetic therapy alone; the second cycle served as the TEAS intervention period, during which patients received chemotherapy and antiemetic therapy plus on-demand TEAS. No randomization or blinding was implemented because of the exploratory self-controlled design. The participant flow through the study is shown in Figure 1. Ethics approval was obtained from the Ethics Committee of Zhejiang Cancer Hospital and The Third Affiliated Hospital of Zhejiang Chinese Medical University (IRB-2025-296(IIT), SLL-KY-2022-042-01). The trial was registered at the Chinese Clinical Trial Registry (ChiCTR2400084368; [registration date: 20240515]) and was conducted according to the approved protocol [protocol version:2.0/date: 20230626] and statistical SOP [version 1.0/20250524].

### 2.2. Diagnostic Criteria

1. Diagnosis of breast cancer confirmed by pathological or cytological examination.
2. Chemotherapy regimen includes emetogenic agents such as anthracyclines, cisplatin, the AC regimen (doxorubicin or epirubicin + cyclophosphamide), or carboplatin (AUC ≥ 4).
3. Presence of nausea, retching, or vomiting during the chemotherapy phase.

### 2.3. Inclusion Criteria

Participants are eligible if they meet all of the following criteria:

1. Met the diagnostic criteria and are aged 20 to 75 years.
2. Experience CINV symptoms during the most recent chemotherapy cycle.
3. Plan to receive at least two consecutive chemotherapy cycles with unchanged chemotherapy and antiemetic regimens in both cycles.
4. Karnofsky Performance Status (KPS) ≥ 60.
5. Expected survival of at least 6 months.
6. Ability to understand study procedures and complete questionnaires.
7. Provide written informed consent.
8. No acupuncture or related therapies (e.g., electroacupuncture, TEAS) within 4 weeks prior to enrollment.

### 2.4. Exclusion Criteria

Participants were excluded if they met any of the following criteria:

1. Concurrent radiotherapy or other anti-cancer therapies during the study period.
2. Gastrointestinal disorders or other medical conditions that may independently cause nausea or vomiting.
3. Severe psychiatric disorders or cognitive impairment that may affect compliance.
4. Skin lesions, infection, or allergy at the PC6 acupoint.
5. Peripheral neuropathy that may interfere with TEAS application.
6. Participation in another clinical trial within 4 weeks prior to enrollment.
7. Inability to comply with study procedures or follow instructions.

### 2.5. Diagnostic and Chemotherapy Rationale

Patients were required to have histologically or cytologically confirmed breast cancer. To reduce heterogeneity in chemotherapy-related emetogenic risk and maintain safety in this exploratory study, eligible regimens primarily included moderately to highly emetogenic chemotherapy, such as anthracycline- and cyclophosphamide-based combinations (e.g., AC and EC), taxane-based regimens (e.g., TC), or other regimens with documented moderate-to-high emetogenic potential, as outlined in clinical guidelines.

At enrollment, participants were scheduled to receive the same chemotherapy and antiemetic regimens during two consecutive cycles. The chemotherapy regimen backbone and emetogenic-risk category remained unchanged between the observation and TEAS intervention periods. The specific antiemetic agents administered during each cycle were obtained from medication administration records. Antiemetic prophylaxis was administered on the day of chemotherapy under the direct supervision of healthcare professionals and verified using these records. During the subsequent 0–120 h follow-up period, no additional self-administered antiemetic use was reported by participants or documented in the study records. Chemotherapy regimen categories, emetogenic-risk categories, and antiemetic prophylaxis categories were summarized for both study periods and are shown in Supplementary Table S1.

The upper age limit of 75 years was retained because of concerns regarding wearable-device usability and limited preliminary safety data in older populations.

### 2.6. Intervention: Patient-Controlled, Real-Time TEAS

#### 2.6.1. Device and Acupoint

**Figure 2:**
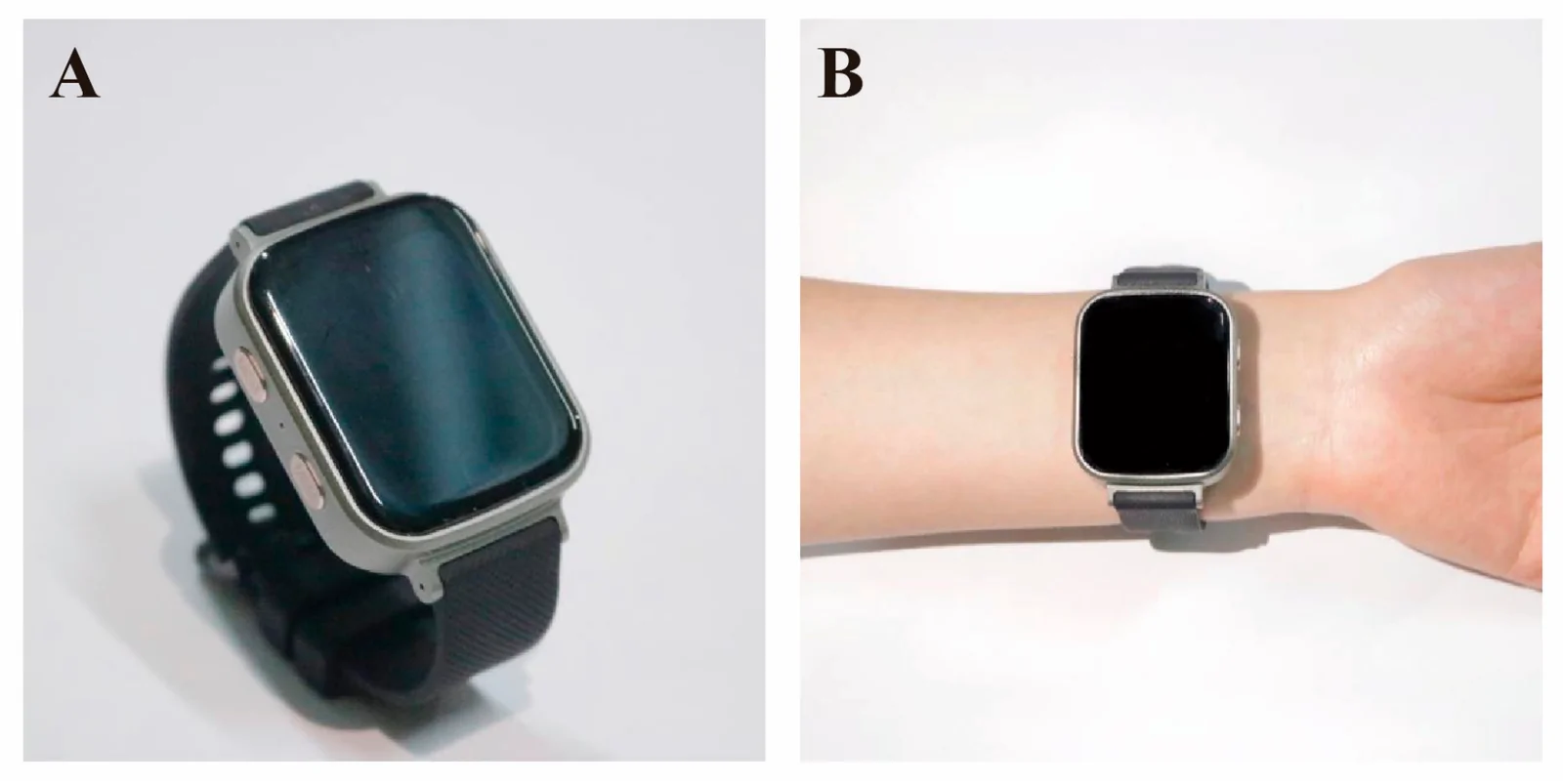
Wrist-type transcutaneous electrical acupoint stimulator and application at PC6. (**A**) Wearable TEAS device. (**B**) Application of the device at Neiguan (PC6) on the forearm. TEAS, transcutaneous electrical acupoint stimulation; PC6, Neiguan.

A wrist-type transcutaneous acupoint electrical stimulator was applied to the Neiguan (PC6), located on the palmar side of the forearm, 2 cun (approximately 6.6 cm) proximal to the wrist crease, between the tendons of the palmaris longus and flexor carpi radialis muscles. The device delivers non-invasive biphasic pulsed electrical stimulation through surface electrodes. The device was developed by Jianxing Zhang and Jiongyao Ye at the School of Information Science and Engineering, East China University of Science and Technology, Shanghai, China. As a self-developed research device, no commercial manufacturer or model number was applicable. The device and its application method are shown in Figure 2.

#### 2.6.2. Device Parameters

The TEAS device delivers biphasic pulsed electrical stimulation in a dense-disperse mode alternating between 2 Hz and 100 Hz. The pulse width was 200 μs. Stimulation intensity was adjusted by the patient to produce a comfortable tingling sensation without pain. Each TEAS session lasted 30 min. If nausea or vomiting persisted, an additional 30 min session could be administered. PC6 was stimulated on one side at a time, with alternating use of the two arms to reduce local discomfort. Stimulation was patient-controlled and could be initiated on demand whenever symptoms occurred.

#### 2.6.3. Intervention Schedule

During the observation period (Cycle 1), patients received standard antiemetic therapy without TEAS; data collected during this cycle served as baseline comparator data.

**Figure 3:**
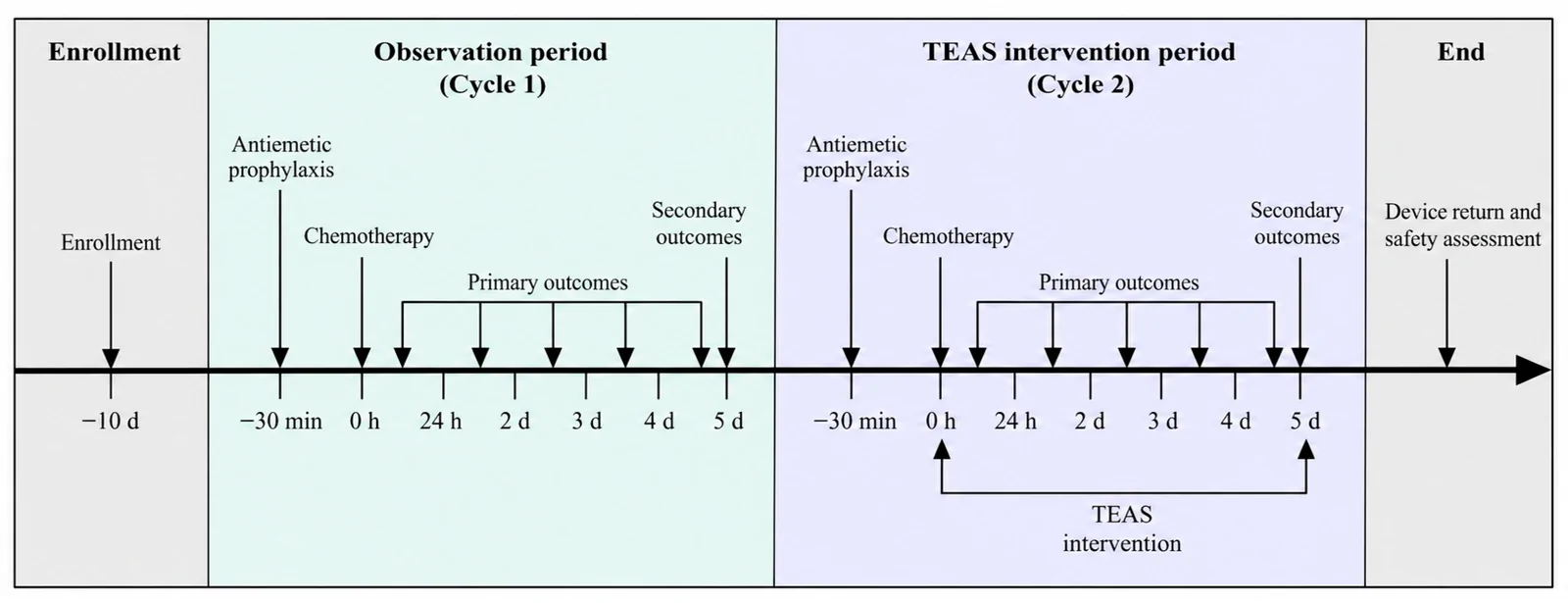
Study timeline. During Cycle 1, patients received standard antiemetic prophylaxis alone, and nausea and vomiting outcomes were recorded during the 0–120 h period after chemotherapy. During Cycle 2, patients received chemotherapy and antiemetic prophylaxis plus patient-controlled, on-demand TEAS initiated at symptom onset during the 0–120 h post-chemotherapy period. Primary outcomes were assessed daily from 24 h to day 5 after chemotherapy, and secondary outcomes were assessed on day 5. At study completion, the device was returned and safety was assessed. The start of chemotherapy is indicated as 0 h. TEAS, transcutaneous electrical acupoint stimulation.

During the TEAS intervention period (Cycle 2), patients self-administered TEAS at symptom onset during the 0–120 h period after chemotherapy. The study process is shown in Figure 3.

Before the intervention, a study researcher instructed participants on device placement and operation. PC6 was identified using anatomical landmarks on the palmar side of the forearm, approximately 2 cun (proximal 6.6 cm) to the wrist crease, between the tendons of the palmaris longus and flexor carpi radialis muscles. The researcher demonstrated the placement procedure before the device was provided for patient-controlled use. After training, participants applied the device independently, and the accuracy of device positioning was not verified during each individual use.

### 2.7. Safety and Monitoring

All TEAS-related adverse events, including skin irritation, local discomfort, numbness, and palpitations, were recorded in real time. Patients were instructed to stop stimulation immediately if discomfort occurred.

Device logs and patient diaries were reviewed daily by study staff. Device logs recorded activation events but did not capture the reason for activation; therefore, they could not distinguish symptom-triggered TEAS from other device-use events.

### 2.8. Outcome Measures

#### 2.8.1. Primary Decision Endpoint and Exploratory Clinical Outcomes

The prespecified binary decision endpoint for Simon’s two-stage design was nausea response on day 1 during the TEAS intervention period, defined as Grade 0 or Grade I. Exploratory clinical outcomes included the frequency and severity of nausea and vomiting at 24 h and on days 2, 3, 4, and 5 after chemotherapy. Symptom severity was assessed using a 0–10 visual analogue scale (VAS), with anchors of 0 = none, 2 = mild, 5 = moderate, 8 = severe, and 10 = unbearable.

#### 2.8.2. Secondary Outcomes

1. Quality of life, assessed using the 36-Item Short Form Health Survey (SF-36), which evaluates physical functioning, role limitations due to physical health, bodily pain, general health, vitality, social functioning, role limitations due to emotional problems, and mental health.
2. Anxiety, measured using the Self-Rating Anxiety Scale (SAS); scores of 50–59, 60–69, and ≥70 indicate mild, moderate, and severe anxiety, respectively.
3. Depression, measured using the Self-Rating Depression Scale (SDS); index scores of <0.50, 0.50–0.59, 0.60–0.69, and ≥0.70 indicate no depression and mild, moderate, and severe depression, respectively.
4. General information, including demographic characteristics, medical history, comorbidities, and medication use.
5. Safety outcomes, including TEAS-related skin irritation, local discomfort, numbness, palpitations, and other adverse events.

### 2.9. Response Evaluation

Treatment response was evaluated according to the criteria from the European Clinical Academic Conference (2019 edition) and the Guiding Principles for Clinical Research of New Chinese Medicines. CINV was graded from 0 to III according to the symptom frequency and impact on daily life. The grading and response evaluation criteria are shown in Table 1. Grades 0 and I were defined as response, and Grades II and III were defined as non-response.

### 2.10. Sample Size Calculation

The sample size was determined using Simon’s two-stage design to minimize clinical risk [24]. Based on prior literature, the null response rate for standard antiemetic therapy was assumed to be approximately 15% [25], and the expected response rate for patient-controlled TEAS was estimated at 30%. In the first stage, 23 evaluable participants were required. If three or fewer participants responded, the study would be stopped for futility. If the study proceeded to the second stage, a total of 48 evaluable participants were required, and if 11 or fewer participants responded after completion of the second stage, the intervention would be considered insufficiently effective. The binary decision endpoint for the Simon two-stage design was nausea response on day 1 during the TEAS intervention period, defined as Grade 0 or Grade I. Dropouts were handled according to the approved protocol, and excluded cases were not included in the final statistical analysis. This design minimized the number of patients exposed while allowing preliminary efficacy to be evaluated.

### 2.11. Statistical Analysis

Normally distributed continuous variables were expressed as mean ± standard deviation (SD) and compared using the paired-sample t test. Non-normally distributed continuous variables were expressed as median (P25, P75) and compared using Wilcoxon signed-rank test. Count data were presented as frequencies (*n*) and percentages (%). A two-sided *p*-value less than 0.05 was considered statistically significant. Statistical analyses were performed using IBM SPSS Statistics, version 19.0 (IBM Corp., Armonk, NY, USA).

Response rates and response categories were summarized descriptively as *n* (%). Paired binary response rates between the observation and TEAS intervention periods were compared using exact McNemar tests. The Simon two-stage decision rule was applied to the prespecified binary response endpoint. Directional changes in symptom grades were summarized using paired patient-day counts and transition matrices; these analyses were exploratory and descriptive.

As a post hoc sensitivity analysis, the paired nausea and vomiting response analyses were repeated after excluding two participants whose antiemetic drug-class combinations differed between the observation and TEAS intervention periods. The same outcome definitions and paired statistical methods used in the full-cohort analysis were applied.

### 2.12. Safety Assessment

Patient safety was monitored throughout the study. Severe adverse events and TEAS-related reactions, such as skin irritation, local discomfort, numbness, or palpitations, were documented in real time. If a patient experienced worsening symptoms or any condition that cannot be relieved by standard treatment and substantially affected daily life, emergency measures could be implemented after specialist assessment. The date, time, type of emergency intervention, and medication use were recorded. A specialist evaluated whether each patient could continue the study.

## 3. Results

### 3.1. Baseline Characteristics

Patient enrollment followed Simon’s two-stage design. The first-stage analysis indicated that more than three responders were observed; therefore, recruitment proceeded to the second stage. A total of 48 patients were enrolled, including 36 from Zhejiang Cancer Hospital, and 12 from the Third Affiliated Hospital of Zhejiang Chinese Medical University. Patient baseline characteristics and clinical features are shown in Table 2. The mean age of the 48 participants was 49.21 ± 9.66 years. The cohort predominantly consisted of patients with stage IV disease, including 1 patient with stage I disease, 4 with stage II disease, 5 with stage III disease, and 38 with stage IV disease.

Most patients received moderately emetogenic chemotherapy, and chemotherapy regimen categories were identical between the two study periods (Supplementary Table S1). Antiemetic prophylaxis was predominantly based on 5-HT3 receptor antagonists. During the observation and TEAS intervention periods, respectively, 13 patients (27.1%) and 13 patients (27.1%) received a 5-HT3 receptor antagonist plus dexamethasone; 11 (22.9%) and 12 (25.0%) received a 5-HT3 receptor antagonist, an NK1 receptor antagonist, dexamethasone, and another antiemetic; and 10 (20.8%) and 11 (22.9%) received a 5-HT3 receptor antagonist, an NK1 receptor antagonist, and dexamethasone. The complete distributions are presented in Supplementary Table S1. The chemotherapy regimen backbone and emetogenic-risk category remained unchanged between the two cycles. Although patients were initially scheduled to receive the same antiemetic regimen, differences in the specific antiemetic agents between periods were identified in five participants. Three involved substitutions within the 5-HT3 receptor antagonist class without a change in the overall drug-class combination, whereas two involved changes in the antiemetic drug-class combination. The reasons for these changes were not documented.

Medication administration records confirmed that the scheduled antiemetic prophylaxis was administered under healthcare supervision on the day of chemotherapy. No additional self-administered antiemetic use was reported or documented during the subsequent 0–120 h assessment period.

### 3.2. Primary and Exploratory Outcome

#### 3.2.1. First-Stage Efficacy Outcomes and Justification for Proceeding to the Second Stage

In the first-stage cohort of 23 patients, the response rate for nausea during the observation period increased from 47.83% on day 1 to 86.96% on day 5 after chemotherapy. During the TEAS intervention period, nausea response rates were 86.96%, 100.00%, 82.61%, 95.65%, and 95.65% on days 1–5, respectively. Compared with the observation period, TEAS was associated with higher nausea response rates at all time points, with absolute improvements of 39.13, 34.78, 13.04, 17.39, and 8.69 percentage points on days 1 to 5, respectively (Supplementary Table S2). The largest improvement was observed on day 1 after chemotherapy. Analysis of response categories further showed that the number of patients with complete response for nausea increased during the TEAS intervention period, particularly from day 3 onward, while the number of patients with no response decreased across all time points (Supplementary Table S3).

For vomiting, the response rate during the observation period was already relatively high, ranging from 86.96% on day 1 to 100.00% on day 3. During the TEAS intervention period, the vomiting response rate reached 100.00% on day 1 after chemotherapy. Compared with the observation period, vomiting response increased on days 1 and 4, remained unchanged on days 2 and 3, and decreased on day 5 (Supplementary Table S4). The number of patients with no response to vomiting was generally low during the TEAS intervention period, although no response was observed in 1 patient on day 2 and 5 patients on day 5 (Supplementary Table S5).

According to the prespecified Simon two-stage design, the study would be terminated after the first stage if the number of day-1 nausea responders was no more than 3, whereas enrollment would proceed if at least 4 responders were observed. In the first-stage cohort, 20 participants met the prespecified day-1 nausea-response criterion during the TEAS intervention period, exceeding the continuation threshold. Therefore, the early stopping criterion was not met, and the study proceeded to the second stage according to the original protocol.

#### 3.2.2. Full-Cohort Outcomes After Completion of the Second Stage

**Figure 4:**
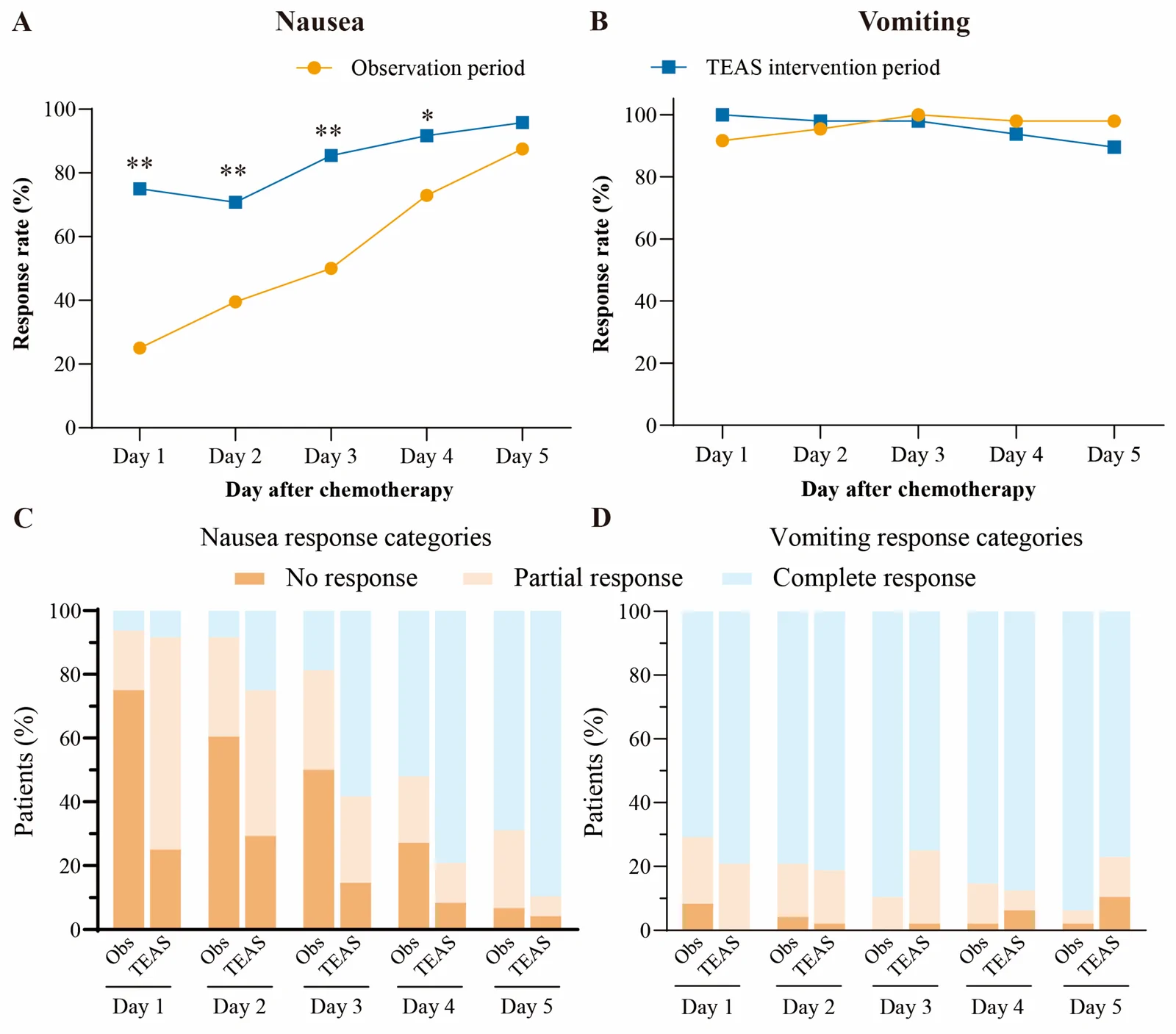
Nausea and vomiting response outcomes during the observation and TEAS intervention periods. Response rates for nausea (**A**) and vomiting (**B**) were compared between the observation and TEAS intervention periods from day 1 to day 5 after chemotherapy. Response-category distributions for nausea (**C**) and vomiting (**D**) are shown as 100% stacked bars. Complete response was defined as Grade 0, partial response as Grade I, and no response as Grade II/III. *p* values were calculated using exact McNemar tests. * *p* < 0.05; ** *p* < 0.01. TEAS, transcutaneous electrical acupoint stimulation; Obs, observation period.

After completion of the second stage, a total of 48 patients were included in the final efficacy analysis. Response was defined as Grade 0 or Grade I, whereas Grade II or Grade III was defined as non-response. For nausea, the response rates were consistently higher during the TEAS intervention period than during the observation period across all five post-chemotherapy days (Figure 4A). The response rates increased from 25.00% to 75.00% on day 1, from 39.58% to 70.83% on day 2, from 50.00% to 85.42% on day 3, from 72.92% to 91.67% on day 4, and from 87.50% to 95.83% on day 5. Exact McNemar tests showed statistically significant paired differences in nausea response rate on days 1–4 after chemotherapy, with *p* < 0.001 on days 1–3 and *p* = 0.012 on day 4, whereas the difference on day 5 was not statistically significant (*p* = 0.125). These findings indicate that the improvement in nausea response was most evident during the first 3 days after chemotherapy and remained statistically significant through day 4 (Table 3).

After excluding the two participants whose antiemetic drug-class combinations differed between periods, the sensitivity analysis included 46 participants. Nausea response rates remained significantly higher during the TEAS intervention period on Days 1–4, whereas the Day 5 difference remained nonsignificant. No significant between-period differences in vomiting response were observed on Days 1–5. The direction and magnitude of the results were consistent with those of the full-cohort analysis (Supplementary Table S6).

Analysis of response categories further showed a shift toward better nausea control during the TEAS intervention period (Figure 4C). The number of patients with complete responses increased, particularly from day 3 onward. Complete response was achieved in 28, 38, and 43 patients on days 3, 4, and 5, respectively, during the TEAS intervention period, compared with 9, 25, and 31 patients during the observation period. In parallel, the number of patients with no response was consistently lower during the TEAS intervention period across all time points.

For vomiting, response rates during the observation period were already high, ranging from 91.67% on day 1 to 100.00% on day 3. During the TEAS intervention period, vomiting response rates ranged from 89.58% to 100.00% (Figure 4B,D). Compared with the observation period, TEAS was associated with small increases in vomiting response rates on days 1 and 2, but response rates were slightly lower during the TEAS intervention period on days 3–5. Exact McNemar tests showed no statistically significant paired differences in vomiting response rate at any post-chemotherapy time point. These results suggest that vomiting was well controlled under standard pharmacological therapy, and no statistically significant additional improvement in vomiting response was observed during the TEAS intervention period.

According to the prespecified final decision rule of the Simon two-stage design, the intervention would be considered to have met the efficacy criterion if more than 11 responders, that is, at least 12 responders, were observed among the 48 patients. In the final analysis, 36 participants met the prespecified day-1 nausea-response criterion during the TEAS intervention period, exceeding the final decision threshold of 12 responders. Therefore, the study met the predefined efficacy criterion after completion of the second stage.

Overall, these results suggest that TEAS, when added to standard pharmacological therapy, was associated with improved control of chemotherapy-induced nausea, particularly during the early post-chemotherapy period. In contrast, no statistically significant between-period difference in vomiting response was observed, probably because vomiting response rates were already high during the observation period.

#### 3.2.3. Paired Changes in Nausea and Vomiting Grades Between the Observation and TEAS Intervention Periods

**Figure 5:**
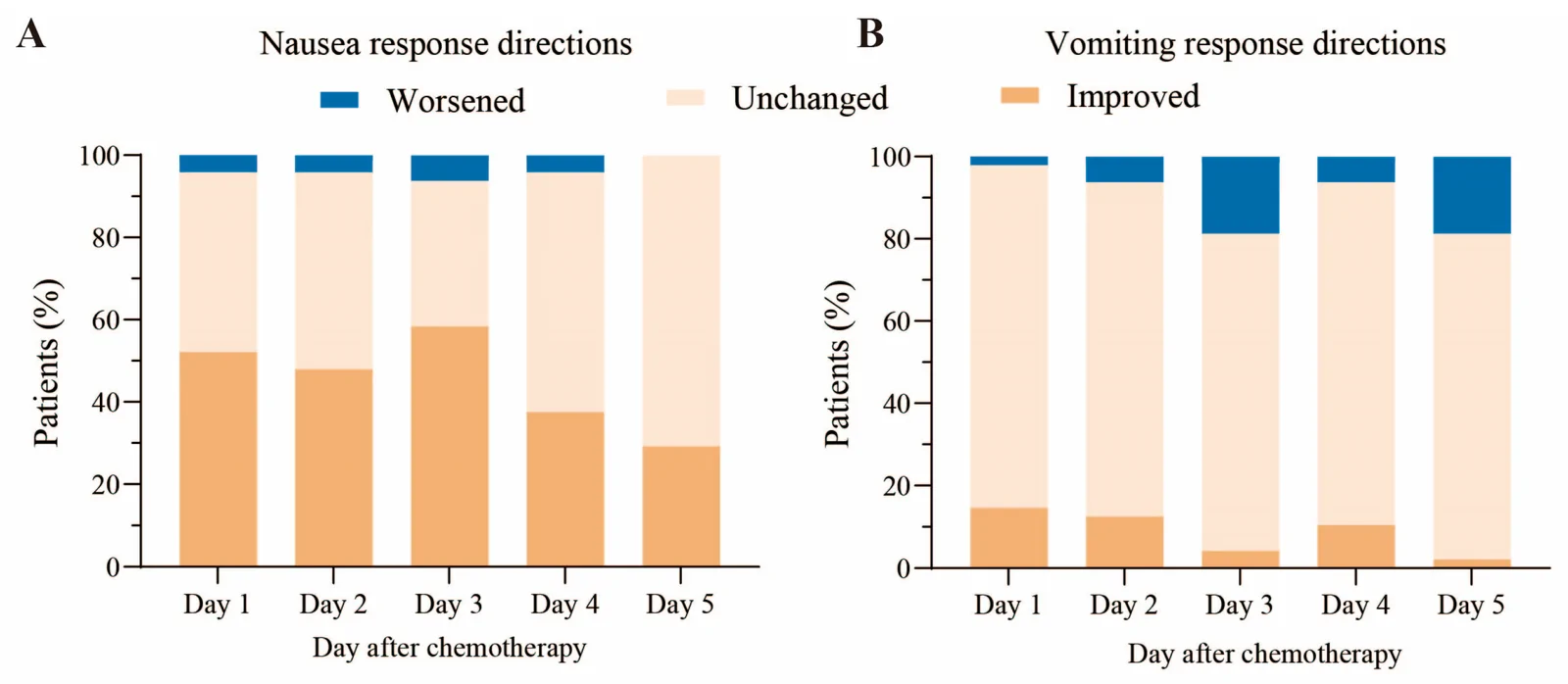
Directional changes in nausea and vomiting during chemotherapy. Panels show the proportions of patient-days with worsened, unchanged, or improved symptoms for nausea (**A**) and vomiting (**B**) from day 1 to day 5 after chemotherapy. Each stacked bar represents the fraction of patients whose symptoms improved, remained unchanged, or worsened with the corresponding observation-period assessment.

To further evaluate whether TEAS was associated with a shift toward lower symptom grades, paired directional changes in nausea and vomiting grades were analyzed across the 5 post-chemotherapy days (Figure 5). For nausea, symptom grades improved in 108 of 240 patient-days (45.00%), remained unchanged in 123 patient-days (51.25%), and worsened in only 9 patient-days (3.75%) during the TEAS intervention period compared with the observation period. The proportion of patients with improved nausea grades was highest on day 3 after chemotherapy (58.33%), followed by day 1 (52.08%) and day 2 (47.92%), suggesting that the favorable association with the TEAS intervention period was most evident during the early post-chemotherapy period.

For vomiting, symptom grades remained unchanged in most patient-days, accounting for 194 of 240 patient-days (80.83%). Improvement was observed in 21 patient-days (8.75%), whereas worsening was observed in 25 patient-days (10.42%). These findings suggest that vomiting grades were largely stable between the two periods, probably because vomiting was already well controlled during the observation period. In contrast, TEAS was associated with a more evident shift toward lower symptom grades for nausea.

#### 3.2.4. Nausea and Vomiting Frequency and Severity During the Observation and TEAS Intervention Periods

**Figure 6:**
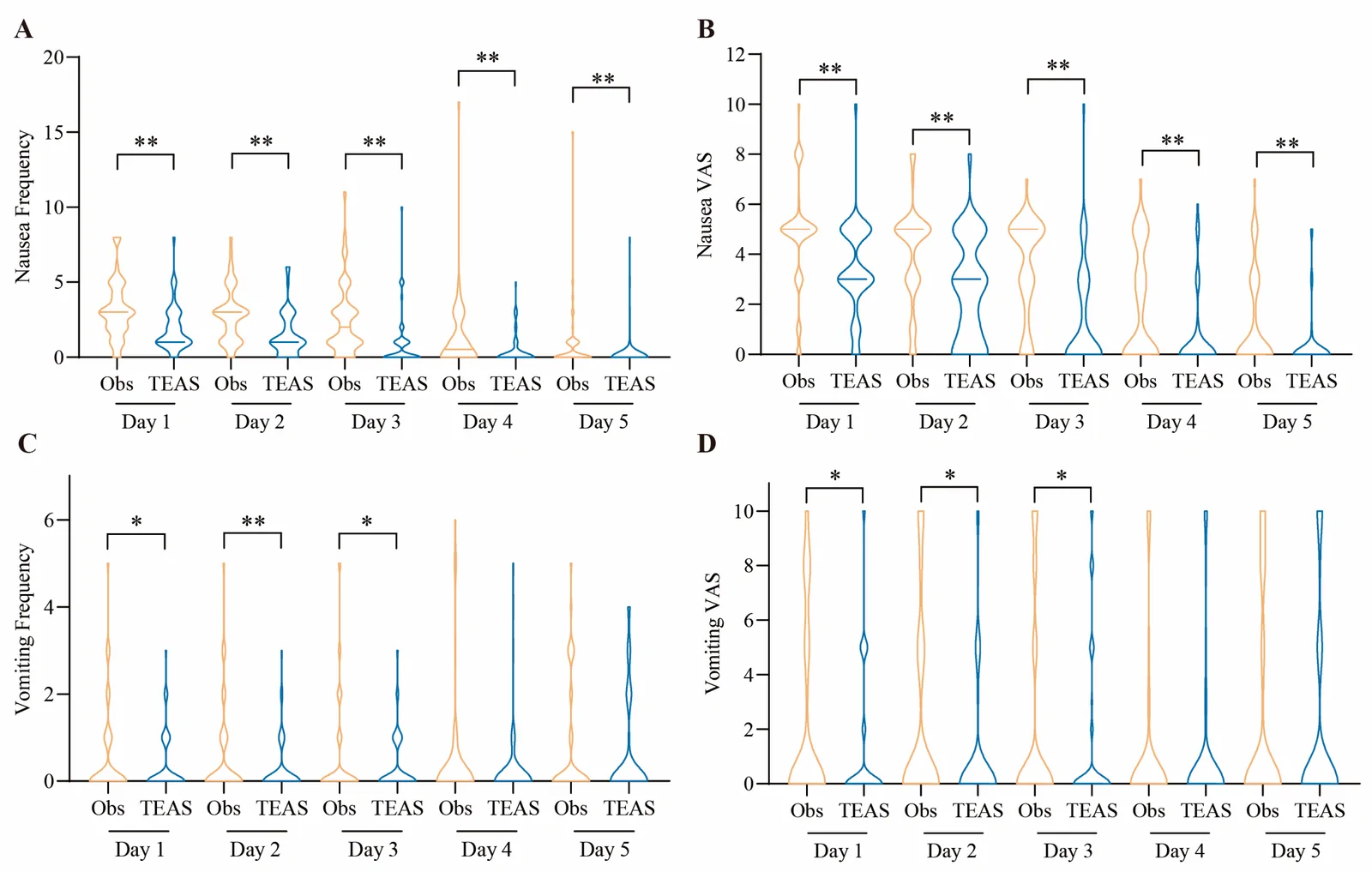
Daily nausea and vomiting frequency and severity during observation and TEAS intervention periods. Violin plots show distributions of nausea frequency (**A**), nausea VAS scores (**B**), vomiting frequency (**C**), and vomiting VAS scores (**D**) from day 1 to day 5 after chemotherapy. Each day includes paired observations from the observation period (Obs) and TEAS intervention period (TEAS). Medians are indicated by dashed lines. Statistical comparisons between periods were performed using Wilcoxon signed-rank tests (* *p* < 0.05; ** *p* < 0.01). TEAS, transcutaneous electrical acupoint stimulation; VAS, visual analogue scale.

During the first 5 days after chemotherapy, both nausea frequency and nausea VAS severity scores were significantly lower during the TEAS intervention period than during the observation period at all time points (Figure 6A,B). The median frequency of nausea decreased from 3.0 episodes in the observation period to 1.0 episode during the TEAS intervention period within the first 24 h after chemotherapy, and from 2.0 episodes to 0 episodes on day 3. Similarly, the median nausea VAS score decreased from 5.0 to 3.0 within the first 24 h and from 5.0 to 0 on day 3 (Figure 6B). These findings indicate that real-time TEAS combined with standard antiemetic therapy was associated with improved control of chemotherapy-induced nausea compared with standard antiemetic therapy alone.

Vomiting outcomes showed a more time-limited pattern. The frequency of vomiting was significantly lower during the TEAS intervention period on days 1, 2, and 3 after chemotherapy (*p* = 0.031, *p* = 0.004, and *p* = 0.025, respectively), whereas no significant difference was observed on days 4 and 5 (Figure 6C). Similarly, vomiting VAS severity scores were significantly lower on days 1–3 (*p* = 0.036, *p* = 0.012, and *p* = 0.046, respectively), but not on days 4 or 5 (Figure 6D). These results indicate between-period differences in vomiting frequency and severity were confined to days 1–3, with no statistically significant differences observed on days 4–5.

### 3.3. Secondary Outcomes

**Figure 7:**
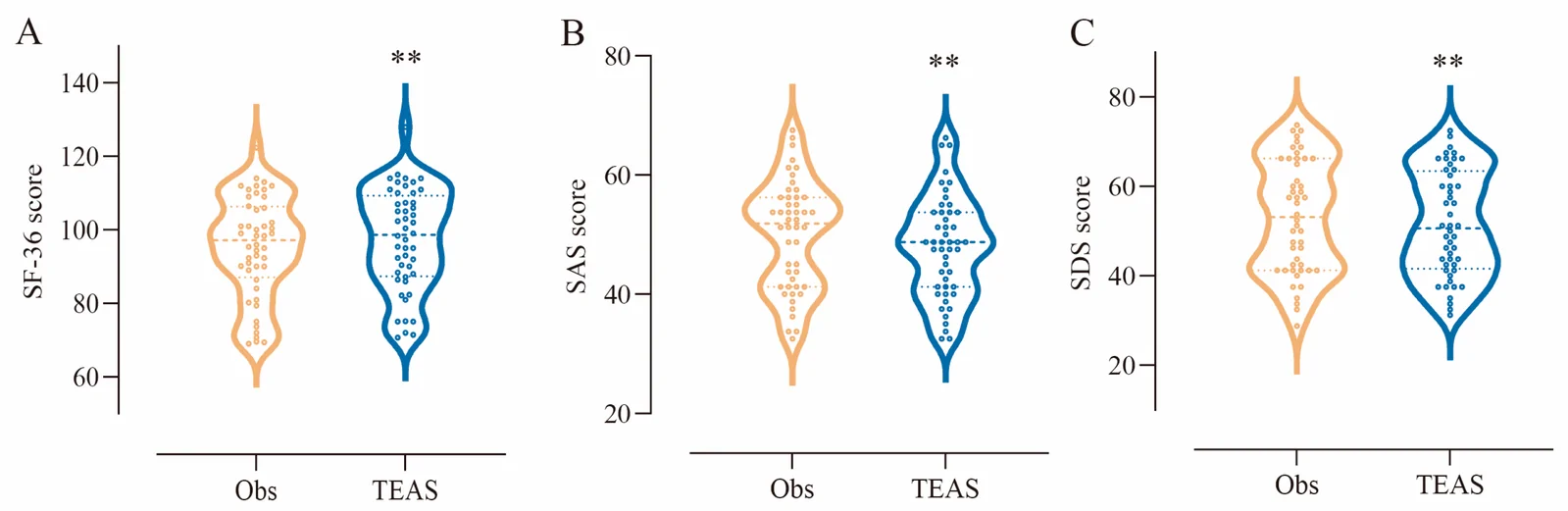
Quality of life, anxiety, and depression scores during the observation and TEAS intervention periods. Violin plots show the distributions of SF-36 scores (**A**), SAS scores (**B**), and SDS scores (**C**) on day 5 after chemotherapy during the observation period (Obs) and TEAS intervention period (TEAS). Medians are indicated by dashed lines. Statistical comparisons between periods were performed using Wilcoxon signed-rank tests. ** *p* < 0.01. TEAS, transcutaneous electrical acupoint stimulation; SF-36, 36-Item Short Form Health Survey; SAS, Self-Rating Anxiety Scale; SDS, Self-Rating Depression Scale.

On day 5, SF-36 score was higher during the TEAS intervention period than during the observation period (Figure 7A, *p* < 0.01). The SAS and SDS scores were lower during the TEAS intervention period (Figure 7B,C, *p* < 0.01). These findings suggest statistically significant but modest improvements in quality of life and emotional status.

### 3.4. Device Utilization and Safety Outcomes

Two patients experienced transient skin numbness at the stimulation site during the TEAS intervention period. Stimulation was temporarily paused and then alternated to the contralateral arm. The numbness gradually subsided and resolved completely without residual sequelae. No serious TEAS-related adverse events occurred.

Device logs confirmed that all participants (48/48, 100%) activated the device at least once during the intervention period. A total of 797 activation events were recorded. The median number of activations per participant was 15 (IQR, 12–19), with a range of 8–44. Because the reason for each activation was not recorded, these events were interpreted as device-use events rather than confirmed symptom-triggered TEAS sessions.

## 4. Discussion

This exploratory study evaluated patient-controlled, real-time TEAS at PC6 as an adjunct to standard antiemetic therapy for CINV in patients with breast cancer. The most consistent finding was improvement in nausea. Compared with the observation period, the TEAS intervention period was associated with lower nausea frequency and severity throughout the 5-day post-chemotherapy observation window, and nausea response rates were higher during the TEAS intervention period, with the most evident differences during the early post-chemotherapy period. The high nausea burden should also be interpreted in light of the eligibility criterion requiring participants to have experienced CINV during their most recent chemotherapy cycle. The cohort was therefore enriched for patients susceptible to CINV and may not be representative of an unselected breast-cancer population.

The response-rate findings should be interpreted in the context of the two-stage design and the small first-stage sample. In the first-stage cohort, the nausea response rate during the observation period was relatively high, particularly on day 1. However, after the planned sample size of 48 patients was completed, the day-1 nausea response rate during the observation period decreased to 25.00%, which was closer to the historical assumption used for sample size estimation and to the range reported in previous studies of standard antiemetic therapy [26]. This pattern suggests that the first-stage estimate may have been inflated by sampling variability. Accordingly, the principal finding should not be interpreted as TEAS efficacy in isolation, but rather as an association between the TEAS intervention period and more favorable nausea outcomes, rather than evidence of isolated TEAS efficacy.

The observed difference in nausea outcomes may be clinically relevant because nausea is often more persistent and more difficult to control than vomiting in routine oncology practice [26]. This difference may reflect distinct neurophysiological mechanisms. Vomiting is a relatively well-characterized reflex coordinated mainly through brainstem pathways, whereas nausea is a more complex subjective experience involving higher central processing, including cortical and limbic circuits [27]. In this context, patient-initiated TEAS may help address an unmet need by providing a timely, nonpharmacological option when nausea begins outside the hospital.

The paired symptom-grade analysis further supports this interpretation. Unlike the response definition used for the Simon design, which combined grades 0 and I as response and grades II and III as no response, the directional grade analysis captured clinically relevant within-category changes. For example, a shift from a higher no-response grade to a lower no-response grade would not change the binary response classification but would still indicate symptom improvement. In the full cohort, nausea grades shifted toward lower severity in 45.00% of patient-days and worsened in only 3.75%, indicating a favorable directional trend during the TEAS intervention period. By contrast, vomiting grades remained unchanged in most patient-days, consistent with the high degree of vomiting control already observed during the standard antiemetic period.

The observed association between real-time TEAS and vomiting outcomes appeared more limited. Reductions in vomiting frequency and severity were observed mainly during days 1–3 after chemotherapy, whereas no significant between-period differences were detected on days 4–5. This pattern may reflect the low number of vomiting events in the later observation window, which reduced the ability to detect between-period differences. It is also possible that delayed vomiting is driven by mechanisms that are less responsive to the stimulation parameters used in this study. These mechanistic interpretations remain speculative and should be tested in larger controlled studies. Although the sensitivity analysis excluding the two participants with changes in antiemetic drug-class combinations yielded findings consistent with the full-cohort analysis, between-period differences in antiemetic treatment remain a potential source of time-varying confounding.

Beyond CINV symptoms, TEAS was associated with statistically significant improvements in SF-36, SAS and SDS scores. These findings are encouraging because CINV can contribute to anxiety, distress, and reduced quality of life. However, the absolute changes were modest, and the clinical significance of these secondary outcomes remains uncertain. Further studies should prespecify minimal clinically important differences and include longer follow-up to determine whether symptom improvements translate into durable quality-of-life benefits.

The patient-controlled design is an important practical feature of this intervention. At enrollment, participants were scheduled to receive the same chemotherapy and antiemetic regimens across two consecutive cycles, an approach intended to reduce major within-patient treatment variability. The wearable wrist-type device also allowed stimulation to be initiated at symptom onset without the need for in-hospital acupuncture administration. This approach may be particularly suitable for outpatient chemotherapy settings, where CINV onset is unpredictable and access to immediate supportive care may be limited.

Safety findings were favorable. Only two patients reported transient local numbness, and both events resolved after temporary interruption and switching stimulation to the opposite arm. No persistent sequelae or serious TEAS-related adverse events were observed. These results suggest that the wearable TEAS device was generally well tolerated in this study population.

### Limitations

This study has several limitations. First, this was a single-arm, self-controlled exploratory trial with a limited sample size; therefore, causal effects cannot be established, and the findings should be interpreted with caution. Period effects between chemotherapy cycles, spontaneous changes in CINV severity, regression to the mean, and expectation effects could not be fully excluded. Second, although the chemotherapy regimen backbone remained unchanged between the two periods, the antiemetic drug-class combination differed in two participants. The sensitivity analysis excluding these participants yielded findings consistent with those of the full-cohort analysis; nevertheless, between-period differences in antiemetic treatment remain a potential source of time-varying confounding. Chemotherapy intensity and antiemetic regimens also varied across participants, contributing to residual clinical heterogeneity. Third, the number of vomiting events was relatively low, particularly during days 4–5, limiting the power to evaluate delayed-phase vomiting. Fourth, because the device was still undergoing functional optimization, TEAS was initiated manually by patients at symptom onset. Although daily follow-up was performed to support adherence and data capture, the timeliness and accuracy of device activation could not be fully guaranteed and may have led to an underestimation or overestimation of the intervention effect. Finally, although changes in SF-36, SAS, and SDS scores were statistically significant, their clinical relevance remains to be established. Because this was an exploratory study, no adjustment for multiplicity was performed, and *p* values should be interpreted descriptively. Although device logs provided objective activation counts, the reason for each activation was not prospectively recorded. We therefore could not determine whether each activation was prompted by nausea or vomiting or whether the device was used for every symptom episode. Consequently, activation frequency should be interpreted as device utilization rather than formal adherence or the exact number of symptom-triggered TEAS sessions. Although participants received instruction on locating PC6 using anatomical landmarks, device placement was not directly observed or objectively verified during each patient-controlled use. Variability in positioning during self-administration therefore could not be excluded. The study could not determine the proportion of activations performed with accurate PC6 placement or assess whether positioning accuracy was associated with treatment outcomes. This uncertainty may have contributed to variability in the observed outcomes. Detailed tumor characteristics, including histological grade, molecular biomarker status, and metastatic sites, were not systematically collected. These variables may influence baseline symptom burden, treatment selection, and the occurrence of nausea or vomiting. Furthermore, most participants had stage IV disease, which limits the generalizability of the findings to patients with early-stage breast cancer. Although patients with medical conditions considered capable of independently causing nausea or vomiting were excluded, residual disease-related confounding could not be completely excluded.

## 5. Conclusions

In this exploratory single-arm clinical trial, patient-controlled, real-time TEAS at PC6 was feasible, well tolerated, and associated with more favorable nausea outcome when added to standard antiemetic therapy in patients with breast cancer. The observed association was most consistent for nausea, whereas between-period differences in vomiting outcomes were mainly observed during the first 3 post-chemotherapy days. These findings support further evaluation of wearable, patient-initiated TEAS in adequately powered randomized, sham-controlled trials, particularly for outpatient management of chemotherapy-induced nausea.

## Figures and Tables

**Table 1:** Grading and evaluation criteria.

Item	Grade 0	Grade I	Grade II	Grade III
Nausea	No nausea	Mild nausea without affecting food intake or daily life	Moderate nausea affecting food intake or daily life	Severe nausea, bedridden due to nausea
Vomiting	No vomiting	1–2 times/day	3–5 times/day	>5 times/day
Efficacy evaluation	Complete response	Partial response	No response	

Grades 0 and I were defined as response; Grades II and III were defined as no response.

**Table 2:** Baseline characteristics of the study participants (*n* = 48).

Item	Result			
Height, cm, mean ± SD (range)	161.3 ± 2.4 (157.0–166.0)			
Weight, kg, mean ± SD (range)	49.5 ± 3.5 (43.6–56.5)			
BMI, kg/m^2^, mean ± SD (range)	18.9 ± 1.1 (17.1–21.2)			
Age, years, mean ± SD (range)	49.21 ± 9.66 (32–74)			
Interval between chemotherapy cycles, days, mean ± SD (range)	19.01 ± 7.77 (6–41)			
KPS score, mean ± SD (range)	74.38 ± 9.43 (60–90)			
Tumor stage *n* (%)	Stage I	Stage II	Stage III	Stage IV
	1 (2.1)	4 (8.3)	5 (10.4)	38 (79.2)

Abbreviations: BMI, body mass index; KPS, Karnofsky performance score.

**Table 3:** Paired analysis of response rates during the observation and TEAS intervention periods in the full cohort.

Outcome	Time	Observation Responders, *n* (%)	TEAS Responders, *n* (%)	Non-Response → Response, *n*	Response → Non-Response, *n*	Difference, Percentage Points	Exact McNemar *p*
Nausea	Day 1	12 (25.00)	36 (75.00)	24	0	50.00	<0.001
Nausea	Day 2	19 (39.58)	34 (70.83)	17	2	31.25	<0.001
Nausea	Day 3	24 (50.00)	41 (85.42)	18	1	35.42	<0.001
Nausea	Day 4	35 (72.92)	44 (91.67)	10	1	18.75	0.012
Nausea	Day 5	42 (87.50)	46 (95.83)	4	0	8.33	0.125
Vomiting	Day 1	44 (91.67)	48 (100.00)	4	0	8.33	0.125
Vomiting	Day 2	46 (95.83)	47 (97.92)	2	1	2.09	1.000
Vomiting	Day 3	48 (100.00)	47 (97.92)	0	1	−2.08	1.000
Vomiting	Day 4	47 (97.92)	45 (93.75)	1	3	−4.17	0.625
Vomiting	Day 5	47 (97.92)	43 (89.58)	0	4	−8.34	0.125

Responders were defined as patients with Grade 0 or Grade I; non-responders were defined as patients with Grade II or Grade III. Non-response → response indicates patients who were non-responders during the observation period but responders during the TEAS intervention period. Response → non-response indicates patients who were responders during the observation period but non-responders during the TEAS intervention period.

## Data Availability

The data presented in this study are available on request from the corresponding author due to privacy and ethical restrictions involving clinical participant data. The study protocol and statistical SOP/statistical analysis plan are available as Supplementary Files.

## Supplementary Materials

The following supporting information can be downloaded at: https://www.mdpi.com/article/10.3390/curroncol33090520/s1, Supplementary Table S1. Summary of chemotherapy regimen categories, emetogenic-risk categories, and antiemetic prophylaxis during the observation and TEAS intervention; Supplementary Table S2. Response outcomes of nausea during the observation and TEAS intervention periods in the first-stage cohort; Supplementary Table S3. Distribution of response categories of nausea during the observation and TEAS intervention periods in the first-stage cohort; Supplementary Table S4. Response outcomes of vomiting during the observation and TEAS intervention periods in the first-stage cohort; Supplementary Table S5. Distribution of response categories of vomiting during the observation and TEAS intervention periods in the first-stage cohort; Supplementary Table S6. Sensitivity analysis of paired nausea and vomiting responses after excluding participants with between-period changes in antiemetic drug-class combinations (*n* = 46); File S2. Protocol version 2.0; File S3. SOP of statistical analysis version 1.0.

## Abbreviations

The following abbreviations are used in this manuscript:

Obs	Observation period
CINV	Chemotherapy-induced nausea and vomiting
TEAS	Transcutaneous electrical acupoint stimulation
