# Supplementary material for: Patient-Controlled Real-Time Transcutaneous Electrical Acupoint Stimulation at Neiguan (PC6) for Chemotherapy-Induced Nausea and Vomiting in Breast Cancer: An Exploratory Single-Arm Two-Stage Trial
Source: Curr Oncol. 2026 Aug 31;33(9):520. doi: 10.3390/curroncol33090520 (PMC13605070; doi:10.3390/curroncol33090520)
Supplement: Supplementary file 1 [file curroncol-33-00520-s001.zip › 1. curroncol-4399059-supplementary table S1-6.pdf]

Supplementary Tabel S1. Summary of chemotherapy regimen categories, emetogenic-risk categories, and antiemetic prophylaxis during the observation and TEAS intervention

Variable	Category	Observation period, n (%)	TEAS intervention period, n (%)
Chemotherapy regimen category	Anthracycline-cyclophosphamide-based	19(39.6)	19(39.6)
	Platinum-containing	13(27.1)	13(27.1)
	Taxane-based	9(18.8)	9(18.8)
	Antibody-drug conjugate-based	3(6.3)	3(6.3)
	Targeted therapy ± chemotherapy	3(6.3)	3(6.3)
	Other regimens	1(2.1)	1(2.1)
Emetogenic-risk category	High emetogenic risk	4 (8.3)	4 (8.3)
	Moderate emetogenic risk	31 (64.6)	31 (64.6)
	Low/minimal emetogenic risk	13 (27.1)	13 (27.1)
Antiemetic prophylaxis category	5-HT3 RA + dexamethasone	13 (27.1)	13 (27.1)
	5-HT3 RA + NK1 RA + dexamethasone + other antiemetic	11 (22.9)	12(25.0)
	5-HT3 RA + NK1 RA + dexamethasone	10 (20.8)	11 (22.9)
	5-HT3 RA + NK1 RA	5 (10.4)	4 (8.3)
	5-HT3 RA only	4 (8.3)	4 (8.3)
	5-HT3 RA + dexamethasone + other antiemetic	4 (8.3)	4 (8.3)
	Dexamethasone + other antiemetic	1 (2.1)	0(0.0)

Data are presented as n (%). Chemotherapy regimen categories were assigned according to the main antineoplastic agents used in each cycle. Emetogenic-risk categories were assigned according to the highest-risk agent within each regimen. Antiemetic prophylaxis categories were summarized according to drug class. 5-HT3 RA, 5-hydroxytryptamine-3 receptor antagonist; NK1 RA, neurokinin-1 receptor antagonist; TEAS, transcutaneous electrical acupoint stimulation.

Supplementary Table S2. Response outcomes of nausea during the observation and TEAS intervention periods in the first-stage cohort

Time after chemotherapy	Observation period: responders, n(%)	TEAS intervention period: responders, n(%)	Difference, n	Difference in response rate, percentage points
Day 1	11(47.83)	20(86.96)	9	39.13
Day 2	15(65.22)	23(100.00)	8	34.78
Day 3	16(69.57)	19(82.61)	3	13.04
Day 4	18(78.26)	22 (95.65)	4	17.39
Day 5	20(86.96)	22(95.65)	2	8.69

Responders were defined as patients with Grade 0 or Grade I. Absolute increase = TEAS intervention period − observation period. Difference in response rate is expressed in percentage points. Denominator was 23 for first-stage analyses.

Supplementary Table S3. Distribution of response categories of nausea during the observation and TEAS intervention periods in the first-stage cohort

Response category	Day 1	Day 2	Day 3	Day 4	Day 5
Observation period					
Complete response	3	3	5	12	14
Partial response	8	12	11	6	6
No response	12	8	7	5	3
TEAS intervention period					
Complete response	18	21	13	19	22
Partial response	2	2	6	3	0
No response	3	0	4	1	1

Complete response = Grade 0; partial response = Grade I; no response = Grade II or III

Supplementary Table S4. Response outcomes of vomiting during the observation and TEAS intervention periods in the first-stage cohort

Time after chemotherapy	Observation period: responders, n(%)	TEAS intervention period: responders, n(%)	Difference, n	Difference in response rate, percentage points
Day 1	20(86.96)	23(100.00)	3	13.04
Day 2	22(95.65)	22(95.65)	0	0.00
Day 3	23(100.00)	23(100.00)	0	0.00
Day 4	22(95.65)	23 (100.00)	1	4.35
Day 5	22(95.65)	18(78.26)	-4	-17.39

Responders were defined as patients with Grade 0 or Grade I. Absolute increase = TEAS intervention period − observation period. Difference in response rate is expressed in percentage points. Denominator was 23 for first-stage analyses.

Supplementary Table S5. Distribution of response categories of vomiting during the observation and TEAS intervention periods in the first-stage cohort

Response category	Day 1	Day 2	Day 3	Day 4	Day 5
Observation period					
Complete response	18	20	21	19	22
Partial response	2	2	2	3	0
No response	3	1	0	1	1
TEAS intervention period					
Complete response	19	18	19	21	16
Partial response	4	4	4	2	2
No response	0	1	0	0	5

Complete response = Grade 0; partial response = Grade I; no response = Grade II or III

Supplementary Table S6. Sensitivity analysis of paired nausea and vomiting responses after excluding participants with between-period changes in antiemetic drug-class combinations (n = 46)

Outcome	Time	Observation responders, n(%)	TEAS responders, n(%)	Non-response → response, n	Response → non-response, n	Difference, percentage points	Exact McNemar *P*
Nausea	Day 1	12 (26.09)	35 (76.09)	23	0	50.00	<0.001
Nausea	Day 2	18 (39.13)	32 (69.57)	16	2	30.43	0.001
Nausea	Day 3	24 (52.17)	39 (84.78)	16	1	32.61	<0.001
Nausea	Day 4	34 (73.91)	42 (91.30)	9	1	17.39	0.021
Nausea	Day 5	40 (86.96)	44 (95.65)	4	0	8.70	0.125
Vomiting	Day 1	42 (91.30)	46 (100.00)	4	0	8.70	0.125
Vomiting	Day 2	44 (95.65)	45 (97.83)	2	1	2.17	1.000
Vomiting	Day 3	46 (100.00)	45 (97.83)	0	1	-2.17	1.000
Vomiting	Day 4	45 (97.83)	43 (93.48)	1	3	-4.35	0.625
Vomiting	Day 5	45 (97.83)	41 (89.13)	0	4	-8.70	0.125

Data are presented as n (%). A responder was defined as a participant with a complete or partial response. Difference represents the TEAS-period response rate minus the observation-period response rate. Exact McNemar tests were based on discordant paired responses. Two participants were excluded because their antiemetic drug-class combinations differed between the observation and TEAS intervention periods.
