# Supplementary material for: Patient-Controlled Real-Time Transcutaneous Electrical Acupoint Stimulation at Neiguan (PC6) for Chemotherapy-Induced Nausea and Vomiting in Breast Cancer: An Exploratory Single-Arm Two-Stage Trial
Source: Curr Oncol. 2026 Aug 31;33(9):520. doi: 10.3390/curroncol33090520 (PMC13605070; doi:10.3390/curroncol33090520)
Supplement: Supplementary file 1 [file curroncol-33-00520-s001.zip › 2. protocol version 2.0.pdf]

Efficacy of Patient-Controlled, Real-Time Transcutaneous Electrical Acupoint Stimulation (TEAS) at Neiguan (PC6) for Chemotherapy-Induced Nausea and Vomiting: A Multicenter, Single-Arm, Two-Stage Clinical Trial Protocol

Huiting Liu[1], Zhou Jie[2], Junying Du[1,3], Qiongying Shen[1], Chao Lu[4], Jianxing Zhang[6], Weiping Zhang[5], Ran Ran[5], Jianqiao Fang[3], Jiongyao Ye[6], Hong Gao[2]* and Junfan Fang[1,3*]

[1]The Third Clinical Medical College, Zhejiang Chinese Medical University, Hangzhou City, China;

[2]Department of Acupuncture and Moxibustion, The Third Affiliated Hospital of Zhejiang Chinese Medical University, Hangzhou City, China;

[3]Key Laboratory of Acupuncture and Neurology of Zhejiang Province, Department of Neurobiology and Acupuncture Research, The Third Affiliated Hospital of Zhejiang Chinese Medical University, Zhejiang Chinese Medical University, Hangzhou, China;

[4]Department of Traditional Chinese Medicine, Zhejiang Cancer Hospital;

[5] Department of Cancer, The Third Affiliated Hospital of Zhejiang Chinese Medical University, Hangzhou City, China;

[6] School of Information Science and Engineering, East China University of Science and Technology, Shanghai, China.

*Corresponding author:

Junfan Fang, E-mail: fangjunfan@163.com

Hong Gao, Email: qtgh@vip.qq.com

ABSTRACT

Introduction Chemotherapy-induced nausea and vomiting (CINV) is one of the most common adverse reactions to chemotherapy. CINV can cause a variety of adverse effects, reducing patients' quality of life and affecting treatment adherence. Pharmacological antiemetics are often insufficient and associated with side effects. Transcutaneous electrical acupoint stimulation (TEAS) at the Neiguan (PC6), a non-invasive form of acupuncture, has demonstrated definitive antiemetic effects. This trial evaluates the efficacy of real-time, patient-controlled TEAS in reducing CINV and improving psychological well-being.

Methods and nalysis This is a multicenter, single-arm, two-stage, self-controlled trial. Patients aged 18-75 years with breast cancer and receiving emetogenic chemotherapy will be enrolled. In Cycle 1, standard antiemetic therapy is applied to establish baseline data. In Cycle 2, patients self-administer TEAS using a portable wristband whenever nausea or vomiting occurs. The primary outcome is nausea and vomiting severity (VAS, 0-10) within 5 days post-chemotherapy. Secondary outcomes include quality of life (SF-36), anxiety (SAS), depression (SDS), and TEAS-related adverse events. Data will be analyzed by comparing observation and trial periods using appropriate statistical methods.

Ethics and Dissemination This clinical trial will be conducted in accordance with the Declaration of Helsinki and has received ethical approval from the Institutional Review Boards of the Third Affiliated Hospital of Zhejiang Chinese Medical University and Zhejiang Cancer Hospital, approval numbers: ZSLL-KY-2022-042-01; IRB-2025-296(IIT). All participants are required to provide written informed consent before enrollment.

Trial Registration Numbers ChiCTR2400084368

Keywords Chemotherapy-induced nausea and vomiting; Transcutaneous electrical acupoint stimulation; *Neiguan* (PC6); Clinical trial protocol; wearable medical devices

Background

Chemotherapy-induced nausea and vomiting (CINV) remains one of the most common and distressing adverse effects of anticancer treatment. The emetogenic potential of the chemotherapy regimen itself is the most critical risk factor for CINV[1]. Most chemotherapy regimens for breast cancer are clearly classified as having moderate-to-high emetic risk. The incidence of CINV after such regimens ranges from 70% to 90%[2]. This indicates that patients with breast cancer undergoing chemotherapy face a very high risk of CINV from the start of treatment. This can lead to dehydration, electrolyte imbalance, and malnutrition[3]. Poorly controlled CINV not only impairs patients' quality of life but may also compromise adherence to chemotherapy, thereby affecting overall treatment outcomes[4]. Although guideline-recommended antiemetic regimens have improved symptom control, up to 20-55.2% of patients still report inadequate relie[5][6]. And these medications are frequently associated with adverse effects such as headache, constipation, and hepatic dysfunction[7]. Therefore, there remains a substantial unmet need for safe and effective complementary approaches to CINV management.

Acupuncture has been widely recommended as a complementary therapy for CINV in both national and international clinical guidelines[8]. Accumulating evidence suggests that acupuncture and related techniques can reduce the incidence and severity of CINV and improve patients' quality of life[9],[10]. Transcutaneous electrical acupoint stimulation (TEAS), a non-invasive modality of acupuncture, has demonstrated efficacy in managing postoperative nausea and vomiting.[11]. In particular, stimulation of the Neiguan (PC6) acupoint has been consistently associated with antiemetic effects.[12]. From a biomedical perspective, TEAS at PC6 may exert its effects through modulation of the autonomic nervous system and central emetic pathways[13], while in traditional Chinese medicine it is believed to regulate qi and harmonize gastrointestinal function.

However, most previous studies have focused on prophylactic, protocol-driven acupuncture interventions, in which treatment is delivered at fixed time points before

or after chemotherapy. In real-world clinical practice, CINV symptoms are often episodic and unpredictable, and their severity varies considerably between patients. As a result, scheduled interventions may not coincide with symptom onset, potentially leading to suboptimal symptom control. Furthermore, standardized treatment regimens do not account for individual variability in symptom burden, limiting the flexibility and responsiveness of acupuncture-based therapies.

To address these limitations, a patient-controlled, real-time intervention strategy may offer a more adaptive approach to symptom management. Allowing patients to self-administer treatment at the onset of nausea or vomiting could improve timeliness of intervention and better match treatment intensity to symptom severity. Based on this rationale, our team has developed a portable, wristband-type TEAS device that enables non-invasive stimulation of the PC6 acupoint. This wearable device is designed to minimize interference with daily activities while supporting on-demand, patient-controlled use.

To date, no studies have specifically evaluated the feasibility and efficacy of patient-controlled, real-time TEAS applied at symptom onset for the management of CINV. Given the exploratory nature of this approach and the lack of preliminary data, we designed a single-arm, two-stage clinical study to assess its feasibility and preliminary efficacy. This study aims to determine whether real-time, patient-controlled TEAS can reduce the severity of CINV and improve patients' psychological well-being and quality of life.

Methods and Design

This was a multicenter, single-arm, two-stage clinical trial evaluating the feasibility and preliminary efficacy of patient-controlled, real-time TEAS as an adjunct to standard antiemetic therapy for CINV. Participants were recruited from Zhejiang Cancer Hospital and The Third Affiliated Hospital of Zhejiang Chinese Medical University. Eligible patients entered two consecutive chemotherapy cycles. The first cycle served as the observation period, during which patients received standard

antiemetic therapy alone; the second cycle served as the TEAS intervention period, during which patients received the same chemotherapy and antiemetic regimens plus on-demand TEAS. No randomization or blinding was implemented because of the exploratory self-controlled design. Screening was conducted according to the approved eligibility criteria. Ethics approval was obtained from the Ethics Committee of Zhejiang Cancer Hospital and The Third Affiliated Hospital of Zhejiang Chinese Medical University (IRB-2025-296(IIT), ZSLL-KY-2022-042-01). The trial was registered at the Chinese Clinical Trial Registry (ChiCTR2400084368). An independent data monitoring committee oversaw the trial conduct and safety. Written informed consent was obtained from all participants before enrollment.

Diagnostic Criteria

1. Diagnosis of breast cancer confirmed by pathological or cytological examination.
2. Chemotherapy regimen includes emetogenic agents such as anthracyclines, cisplatin, the AC regimen (doxorubicin or epirubicin + cyclophosphamide), or carboplatin (AUC ≥ 4).
3. Presence of nausea, retching, or vomiting during the chemotherapy phase.

Inclusion Criteria

Participants are eligible if they meet all of the following criteraia:

1. Met the diagnostic criteria and are aged 20 to 75 years.
2. Experience CINV symptoms during the most recent chemotherapy cycle.
3. Plan to receive at least two consecutive chemotherapy cycles with unchaged chemotherapy and antiemetic regimens in both cycles.
4. Karnofsky Performance Status (KPS)≥60.
5. Expected survival of at least 6 months.
6. Ability to understand study procedures and complete questionnaires.
7. Provide written informed consent.
8. No acupuncture or related therapies (e.g., electroacupuncture, TEAS) within 4 weeks prior to enrollment.

Exclusion Criteria

Participants were excluded if they met any of the following criteria:

1. Concurrent radiotherapy or other anti-cancer therapies during the study period.
2. Gastrointestinal disorders or other medical conditions that may independently cause nausea or vomiting.
3. Severe psychiatric disorders or cognitive impairment that may affect compliance.
4. Skin lesions, infection, or allergy at the PC6 acupoint.
5. Peripheral neuropathy that may interfere with TEAS application.
6. Participation in another clinical trial within 4 weeks prior to enrollment.
7. Inability to comply with study procedures of follow instructions.

Diagnostic and Chemotherapy Rationale

Patients were required to have histologically or cytologically confirmed breast cancer. To reduce heterogeneity in chemotherapy-related emetogenic risk and maintain safety in this exploratory study, the trial primarily includes patients receiving moderately to highly emetogenic chemotherapy. Typical eligible regimens include anthracycline- and cyclophosphamide-based combinations (e.g., AC, EC, TC) or other regimens with documented moderate to high emetogenic potential, as outlined in clinical guidelines. The upper age limit of 75 years was retained because of concerns regarding wearable-device usability and limited preliminary safety data in older populations.

Intervention: Patient-Controlled, Real-Time TEAS

Device and Acupoint

A wrist-type transcutaneous acupoint electrical stimulator was applied to the Neiguan (PC6), located on the palmar side of forearm, 2 cun (approximately 6.6 cm) proximal to the wrist crease, between the tendons of the palmaris longus and flexor carpi radialis muscles. The device delivers non-invasive biphasic pulsed electrical stimulation through surface electrodes.

Device Parameters

The TEAS device delivers biphasic pulsed electrical stimulation in a dense-dispersed mode alternating between 2 Hz and 100 Hz. The pulse width is set at 200 microseconds, and the stimulation intensity is adjusted by the patient to a comfortable tingling sensation without causing pain. Each TEAS session lasts 30 minutes, and if nausea or vomiting persists, an additional 30-minute session may be applied.

Stimulation is continuous during the session and is patient-controlled, allowing on-demand application whenever symptoms occur.

Intervention Schedule

During the observation period (Cycle 1), patients received standard antiemetic therapy without TEAS, data collected during this cycle served as baseline comparator data. During the TEAS intervention period (Cycle 2), patients self-administered TEAS at symptom onset during the 0–120 h period after chemotherapy. Chemotherapy and antiemetic therapy remained unchanged between the two cycles.

Safety and Monitoring

All TEAS-related adverse events, including skin irritation, discomfort, or palpitations, are recorded in real time. Patients are instructed to stop stimulation immediately if discomfort occurs. Device logs and patient diaries are reviewed daily by study staff.

Adherence

The device automatically records usage time and intensity. Patients also maintain a daily diary of nausea severity, vomiting episodes, and TEAS usage.

Outcome Measures

Primary Outcome

The primary outcome were the frequency and severity of nausea and vomiting at 24 hours and on day 2, 3, 4 and 5 after chemotherapy. Symptom severity was assessed using a 0–10 Visual Analogue Scale (VAS), with anchors of 0 = none, 2 = mild, 5 = moderate, 8 = severe, 10 = unbearable.

Secondary Outcomes

1. Quality of life, assessed using the 36-Item Short Form Health Survey (SF-36), which evaluates physical functioning, role limitations due to physical health, bodily pain, general health, vitality, social functioning, role limitations due to emotional problems, and mental health.

2. Anxiety, measured using the Self-Rating Anxiety Scale (SAS); scores of 50–59, 60–69, and ≥70 indicate mild, moderate, and severe anxiety, respectively.

3. Depression, measured using the Self-Rating Depression Scale (SDS); index scores of <0.50, 0.50–0.59, 0.60–0.69, and ≥0.70 indicate no depression and mild, moderate,

and severe depression, respectively.

4. General information, including demographics characteristics, medical history, comorbidities, and medication use.

5. Safety outcomes, including TEAS-related skin irritation, local discomfort, numbness, palpitations, and other adverse events.

Sample Size Calculation

The sample size was determined using Simon's two-stage design to minimize clinical risk[14] Based on prior literature, the null response rate for standard antiemetic therapy is approximately 15%[15], while the expected response rate for patient-controlled TEAS is estimated at 30%. In the first stage, 23 subjects will be enrolled, and if three or fewer respond, the study will be terminated for futility. In the second stage, the total number of subjects will increase to 48, and if 11 or fewer respond, the study will be terminated. Dropouts during the study will be included in the analysis according to the protocol, while excluded cases will not be counted in the statistical analysis. This approach ensures that the minimum number of patients is enrolled to evaluate preliminary efficacy while reducing exposure to an ineffective intervention.

Statistical Analysis

All statistical analyses will be conducted by an independent statistician who is not involved in the clinical trial. Continuous variables will be described using mean ± standard deviation, median, minimum, maximum, and quartiles, while categorical variables will be expressed as percentages. or continuous data following a normal distribution, descriptive statistics are expressed as mean ± standard deviation ($\bar{x} \pm s$), and comparisons before and after treatment are performed using the paired-samples t-test. For continuous data not following a normal distribution, descriptive statistics are expressed as median (lower quartile, upper quartile) [M (P25, P75)], and comparisons are performed using the Wilcoxon signed-rank test. All tests are two-sided, with a significance level set at $\alpha = 0.05$. $P < 0.05$ is considered statistically significant. For subgroup comparisons, appropriate statistical methods will be applied based on data type and distribution, including one-way ANOVA or Kruskal-Wallis test

for continuous variables, chi-square or Fisher's exact test for categorical variables, and Kruskal-Wallis or Cochran-Mantel-Haenszel tests for ordinal variables. Categorical data are expressed as frequency (n) and percentage (%) for descriptive statistics. All analyses will be completed promptly after data entry and verification, and a written statistical report will be issued by the statistician.

Safety Assessment

Patient safety will be monitored throughout the study. Any severe adverse events or TEAS-related reactions, such as skin irritation, discomfort, or palpitations, will be documented in real time. If a patient experiences worsening disease symptoms or any condition that cannot be relieved by standard treatment and significantly affects daily life, emergency measures may be implemented after specialist assessment. The date, time, and type of any emergency intervention, including medication use, will be recorded. The specialist will evaluate whether continuation of the study is appropriate for each patient.

Discussion

Nausea and vomiting are common clinical symptoms observed in a variety of diseases, and CINV represents a special type arising primarily from the body's response to cytotoxic drugs. The physiological mechanisms underlying nausea remain incompletely understood, as it is predominantly a subjective experience reported by patient[16]. In contrast, vomiting is better characterized and is largely mediated by the cytotoxicity of chemotherapeutic agents, such as platinum-based drugs. These agents interact with 5-hydroxytryptamine type 3 (5-HT3), neurokinin 1 (NK1), and dopamine D2/D3 receptors in the gastrointestinal tract, either directly or via neurotransmitter release[17]-[19]. Signals are then transmitted through the gut-brain axis to the dorsal vagal complex in the brainstem, triggering the emetic reflex[20]. Currently, antiemetic medications are the primary of CINV; however, their adverse effects can be significant. Acupuncture, by contrast, has gained increasing attention for its non-invasive, low-risk, and patient-friendly properties. TEAS as a novel from of acupuncture, addresses several limitations of traditional acupuncture, including

high technical requirements, challenges in clinical coordination, and lower patient acceptance. In this study, the *Neiguan* (PC6) acupoint was selected for stimulation. Its antiemetic mechanism is believed to involve modulation of the neuroendocrine system, producing a broad regulatory effect. Prior studies have shown that TEAS at PC6 can significantly reduce serum 5-HT and dopamine levels, suggesting that its antiemetic effect may be mediated via serotonin- and dopamine-related pathways[21]. Other research indicates that the antiemetic action of PC6 is closely associated with vagus nerve regulation[22].

If this trail demonstrates that real-time, patient-controlled TEAS as PC6 effectively reduces the frequency and severity of nausea and vomiting, alleviates anxiety and depression, and improves quality of life, it could substantially enhance the supportive care of cancer patients and complement pharmacological therapies. The design of this study incorporates several strengths:

Real-time intervention: Acupuncture efficacy is influenced by multiple factors, including needle insertion parameters, stimulation intensity, intervention timing, retention time, and treatment frequency[23][24]. Previous RCTs, such as those by Wang, have shown that pre-chemotherapy acupuncture may provide greater benefit for CINV[25]. However, it remains unclear whether real-time, symptom-triggered intervention is more effective in highly episodic conditions like CINV. This study emphasizes patient-controlled, real-time TEAS, enabling stimulation at the precise onset of nausea or vomiting. Conventional acupuncture is limited by human resources and scheduling, often resulting in mismatches between treatment and symptom onset. The portable TEAS wristband used in this trial allows patients to self-administer stimulation when symptoms occur, ensuring timely and effective intervention.

High patient compliance: The TEAS device is non-invasive, lightweight, and minimally disruptive to daily activities, allowing prolonged wear and flexible use. Patients can adjust the duration of stimulation according to symptom severity, which enhances both acceptance and adherence to the therapy. These factors are expected to improve therapeutic outcomes compared with conventional or scheduled acupuncture.

Cost-effectiveness and ethical efficiency: The trial employs a single-arm, two-stage

design. In the first stage, 23 participants are recruited, and if three or fewer show response, the study is terminated. Otherwise, it proceeds to the second stage with a total of 48 participants; if 11 or fewer respond, the study is stopped. This design minimizes resource waste and ethical concerns by limiting patient exposure to ineffective interventions. Additionally, the reusable TEAS wristbands reduce environmental impact and overall study costs.

Research significance and future directions: This study not only evaluates the efficacy and safety of real-time, patient-controlled TEAS for CINV but also provides a model for flexible, patient-driven supportive care interventions in oncology. If successful, the approach could be expanded to other episodic chemotherapy-related symptoms and chronic nausea conditions, and inform the design of wearable, self-administered therapies. Furthermore, insights from this trial may contribute to understanding the neurophysiological mechanisms of acupuncture and support the integration of non-pharmacological interventions into routine clinical practice, improving overall patient well-being and treatment adherence.

Reference

[1] Herrstedt J, Clark-Snow R, Ruhlmann CH, et al. 2023 MASCC and ESMO guideline update for the prevention of chemotherapy- and radiotherapy-induced nausea and vomiting[J]. ESMO Open, 2024, 9(2): 102195.

[2] Piechotta V, Adams A, Haque M, et al. Antiemetics for adults for prevention of nausea and vomiting caused by moderately or highly emetogenic chemotherapy: a network meta-analysis[J]. Cochrane Database of Systematic Reviews, 2021(11): CD012775

[3] Heckroth M, Luckett RT, Moser C, Parajuli D, Abell TL. Nausea and Vomiting in 2021: A Comprehensive Update. J Clin Gastroenterol. 2021;55(4):279-299.

[4] Gupta K, Walton R, Kataria SP. Chemotherapy-Induced Nausea and Vomiting: Pathogenesis, Recommendations, and New Trends[J]. Cancer Treatment and Research Communications, 2021, 26: 100278.

[5] Ng B, Astari YK, Adrian Wiranata J, et al. Chemotherapy-induced nausea and vomiting in patients with breast cancer: risk factor and predictive model using classification and regression tree (CART)[J]. Cureus, 2023, 15(8): e44438.

[6] Grunberg S M, Deuson R R, Mavros P, et al. Incidence of chemotherapy-induced nausea and emesis after modern antiemetics[J]. Cancer, 2004, 100(10): 2261-2268.

[7] Sun H, Zhang X, Xin X, et al. Efficacy and Safety of Different 5-HT3 Receptor Antagonists as Monotherapy for Preventing Nausea and Vomiting in Patients Undergoing Surgery or Chemotherapy: A Network Meta-Analysis of Randomized Controlled Trials. J Clin Pharmacol. 2025;65(11):1587-1597.

[8] Mariani S, Quagliariello V, Bertocchi P, et al. Integrating nutrition, physical exercise, psychosocial support and antiemetic drugs into CINV management: The road to success[J]. Cancer Treatment Reviews, 2024, 129: 102798.

[9] Rithirangsriroj K, Manchana T, Akkayagorn L. Efficacy of acupuncture in prevention of delayed chemotherapy induced nausea and vomiting in gynecologic cancer patients. Gynecol Oncol. 2015; 136(1): 82-86.

[10] Lin R, Guo Z, Zhang J, et al. Acupuncture for nausea and vomiting induced by highly emetogenic chemotherapy:a systematic review and meta-analysis[J]. Frontiers in Neurology, 2026, 16: 1692411.

[11] Lu L, Xie C, Li X, et al. Efficacy and safety of electrical acupoint stimulation for postoperative nausea and vomiting: A systematic review and meta-analysis[J]. PLoS ONE, 2023, 18(5): e0285943.

[12] Xiao L;Chen Z;Jianfei S. Acupoint selection rules in treatment of chemotherapy-I nduced nausea and vomiting with acupuncture and Moxibustion. World Journal of Acupuncture-Moxibustion 2022, 32 (2), 149 – 156.

[13] Zhang F, Cai R, Yu Q, et al. Electroacupuncture suppresses premature ventricular complexes occurring post-myocardial infarction through corticothalamic circuit. Cell Rep. 2025;45(11):116734.

[14] Xiang M, Ma Y, Li Q. Two-Stage Design Sample Size Determination for Two Doses in Oncology Phase II Trials[J]. The New England Journal of Statistics in Data Science, 2025, 3(3): 253-264

[15] Sun DS, Ko YH, Jin JY, et al. Efficacy of the granisetron transdermal system for the control of nausea and vomiting induced by highly emetogenic chemotherapy: a multicenter, randomized, controlled trial[J]. The Korean Journal of Internal Medicine, 2023, 38(3): 406-416.

[16] Bo š njak SM, Gralla RJ, Schwartzberg L. Prevention of chemotherapy-induced nausea: the role of neurokinin-1 (NK) receptor antagonists. Support Care Cancer. 2017 May;25(5):1661-1671.

[17] Miao R, Jiang C, Chang WY, et al. Gasdermin D permeabilization of mitochondrial inner and outer membranes accelerates and enhances pyroptosis[J]. Immunity, 2023, 56(11): 2523-2541.e8.

[18] Stoica S, Hogge C, Theeler B J. Refractory nausea and vomiting due to central nervous system injury: a focused review[J]. Life (Basel), 2025, 15(7): 1021.

[19] Belkacemi L, Darmani N A. Dopamine receptors in emesis: Molecular mechanisms and potential therapeutic function[J]. Pharmacological Research, 2020, 161: 105124.

[20] Ullah I, Ayaz M. A re-consideration of neural/receptor mechanisms in chemotherapy-induced nausea and vomiting: current scenario and future perspective[J]. Pharmacological Reports, 2023, 75(5): 1126-1137.

[21] Zhang X, Jin H F, Fan Y H, et al. Effects and mechanisms of transcutaneous electroacupuncture on chemotherapy-induced nausea and vomiting[J]. Evidence-Based Complementary and Alternative Medicine, 2014, 2014: 860631.

[22] Mao T, Cheng Q, Liu X, et al. Effect of electrical stimulation on gastrointestinal symptoms in lung cancer patients during chemotherapy: a randomized controlled trial[J]. Asia-Pacific Journal of Oncology Nursing, 2021, 8(3): 246-254.

[23] Qu F, Zhang GR. Transcutaneous electrical acupoint stimulation: a potential bridge linking traditional Chinese medicine and AI-driven transformation in modern healthcare[J]. Integrative Medicine Research, 2025, 14(3): 101221.

[24] Chaoda L;Chenyang Q;Boxuan L;Shizhe D; Zhihong M. Research Progress of Time,Frequency,Direction and Depth in Acupuncture Manipulation Metrology. Journal of Clinical Acupuncture and Moxibustion 39 (2), 105–110

[25] Yaling W;Jinxia l;Xiaoqing G;Guiyang F;Xinjun G. Effect of Acupuncture Intervention at Different Time on Nausea and Vomiting Induced by Chemotherapy for Lung Cancer. Chinese Acupuncture & Moxibustion.**2019**, *39* (12), 1269–1273.

Acknowledgements

We would like to thank the patients and health professionals for their contributions throughout the development of the interventions.

Author contributors

JF(Junfan Fang) and JD designed the study and interventions. HL and HG has contributed to the paper in terms of research, planning and writing the protocol. JZ and JY designed the wearable TEAS device. QS, JZ, CL, HL ,RR and WZ conduct the clinical studies. JF and JF(Jianqiao Fang) provide the funding.

Funding

This research was funded by the Key project of Zhejiang Provincial Administration of Traditional Chinese Medicine, grant number GZY-ZJ-KJ-23072, National Key R&D Program of China, grant number 2022YFC3500404, the National Natural Science Foundation of China, grant number 82174490 and 82374561.

Competing interests

The authors declare that they have no competing interests in this work. Patient and public involvement Patients and/or the public were involved in the design, or conduct, or reporting, or dissemination plans of this research. Refer to the Methods section for further details.

Patient consent for publication Applicable.
