# Supplementary material for: Patient-Controlled Real-Time Transcutaneous Electrical Acupoint Stimulation at Neiguan (PC6) for Chemotherapy-Induced Nausea and Vomiting in Breast Cancer: An Exploratory Single-Arm Two-Stage Trial
Source: Curr Oncol. 2026 Aug 31;33(9):520. doi: 10.3390/curroncol33090520 (PMC13605070; doi:10.3390/curroncol33090520)
Supplement: Supplementary file 1 [file curroncol-33-00520-s001.zip › 3. SOP of statistical analysis version 1.0. .pdf]

Standard Operating Procedure for Statistical Analysis

Patient-Controlled Real-Time Transcutaneous Electrical Acupoint Stimulation at Neiguan (PC6) as an Adjunct to Antiemetics for Chemotherapy-Induced Nausea and Vomiting in Breast Cancer

Document Control

**SOP title**	Standard Operating Procedure for Statistical Analysis
**Study title**	Patient-Controlled Real-Time Transcutaneous Electrical Acupoint Stimulation at Neiguan (PC6) as an Adjunct to Antiemetics for Chemotherapy-Induced Nausea and Vomiting in Breast Cancer: A Single-Arm Two-Stage Trial
**Trial registration**	ChiCTR2400084368
**Ethics approval numbers**	ZSLL-KY-2022-042-01; IRB-2025-296(IIT)
**SOP version / date**	Version 1.0 / 2025-04-25
**Prepared by**	Huiting Liu, The Third Clinical Medical College, Zhejiang Chinese Medical University
**Reviewed by**	Prof. Junfan Fang, The Third Clinical Medical College, Zhejiang Chinese Medical University
**Approved by**	Prof. Junfan Fang, The Third Clinical Medical College, Zhejiang Chinese Medical University

1. Purpose

This Standard Operating Procedure (SOP) defines the statistical analysis workflow, analysis populations, endpoint derivations, statistical methods, quality-control procedures, documentation requirements, and approval process for the above clinical study. The SOP is intended to support reproducibility, traceability, and consistency between the clinical database, statistical outputs, manuscript tables, and reported results.

2. Scope

This SOP applies to all statistical analyses performed for the clinical manuscript and any associated supplementary materials, including efficacy, response-rate, paired symptom-grade, secondary outcome, and safety analyses. It covers analyses performed after completion of the first-stage cohort and after completion of the full cohort.

This SOP does not replace the approved study protocol, ethics approval documents, or clinical trial registration record. If there is any discrepancy, the approved protocol and ethics requirements take precedence, and any deviation must be documented in the analysis deviation log.

3. Study Overview

The study is a multicenter, single-arm, two-stage exploratory clinical trial with a self-controlled design. Participants received two consecutive chemotherapy cycles with unchanged chemotherapy and antiemetic regimens. The first cycle served as the observation period, during which standard antiemetic therapy alone was used. The second cycle served as the TEAS intervention period, during which standard antiemetic therapy plus patient-controlled, on-demand TEAS at PC6 was used.

The intervention window was 0-120 hours after chemotherapy. Outcomes were assessed at 24 hours and on days 2, 3, 4, and 5 after chemotherapy. All 48 enrolled patients were included in the final efficacy analysis and safety assessment, provided that they completed the required paired observations according to the approved protocol.

4. Roles and Responsibilities

Role	Responsibilities
**Principal investigator / corresponding author**	Overall responsibility for study conduct, approval of the statistical analysis SOP, interpretation of results, and final approval of submitted materials.
**Clinical investigators**	Ensure accurate source data collection, completion of case report forms and patient diaries, documentation of protocol deviations and safety events, and resolution of clinical data queries.
**Data manager / designated study staff**	Perform data entry, data cleaning, consistency checks, coding of categorical variables, database lock, and maintenance of the data query and change logs.
**Statistician / statistical analyst**	Implement the statistical methods specified in this SOP, generate reproducible outputs, maintain analysis syntax/scripts, document deviations, and perform consistency checks between outputs and manuscript tables.
**Independent reviewer / quality-control reviewer**	Review selected datasets, statistical code, output tables, and manuscript values against source outputs before submission.

5. Source Data and Data Management

5.1 Data sources. Statistical analyses shall use data derived from the approved case report forms, patient daily diaries, symptom grading records, VAS records, SF-36, SAS, SDS forms, adverse-event records, and device-related records when applicable.

5.2 Data structure. The analysis dataset shall include one record per participant for baseline and safety summaries and one record per participant per assessment day for repeated symptom outcomes. Variables shall identify participant ID, study center, assessment day, observation-period values, TEAS-intervention-period values, response category, binary response status, symptom frequency, VAS score, and safety events as applicable.

5.3 Data cleaning. Before analysis, the data manager and statistician shall perform range checks, logical checks, paired-data completeness checks, consistency checks between symptom grades and binary response classification, and verification of any extreme or inconsistent values against the source record.

5.4 Database lock. Statistical analysis shall be conducted only after the analysis dataset is finalized or locked. Any post-lock data correction must be documented with the reason, date, responsible person, affected variables, and impact on outputs.

6. Analysis Populations

Analysis population	Definition
**Full efficacy analysis set**	All enrolled participants who completed the required paired observation-period and TEAS-intervention-period assessments and were included in the final efficacy analysis. In the manuscript, this set includes 48 patients.
**First-stage analysis set**	The first 23 evaluable participants analyzed according to Simon two-stage design rules to determine whether the study should proceed to the second stage.
**Safety analysis set**	All participants who received or attempted TEAS during the TEAS intervention period and had safety follow-up. TEAS-related adverse events are summarized descriptively.

7. Endpoint Definitions and Derived Variables

7.1 Symptom grading. Nausea and vomiting are graded from Grade 0 to Grade III according to the grading and evaluation criteria used in the manuscript. Lower grades indicate less severe symptoms.

7.2 Binary response. For response-rate analyses, Grade 0 and Grade I are defined as response; Grade II and Grade III are defined as non-response.

7.3 Directional grade change. For each participant and assessment day, grade change is classified as improved, unchanged, or worsened. Improvement is defined as a lower symptom grade during the TEAS intervention period than during the observation period. Worsening is defined as a higher symptom grade during the TEAS intervention period than during the observation period. Unchanged indicates the same grade in both periods.

7.4 Frequency and VAS outcomes. Daily frequency of nausea and vomiting episodes and VAS severity scores are analyzed as paired continuous or ordinal-like outcomes. VAS is scored from 0 to 10, with higher scores indicating more severe symptoms.

7.5 Secondary outcomes. SF-36, SAS, and SDS scores are analyzed as paired outcomes comparing the observation period and TEAS intervention period at day 5, as specified in the manuscript.

7.6 Safety outcomes. Safety outcomes include TEAS-related skin irritation, local discomfort, numbness, palpitations, and other adverse events recorded during the study. Serious adverse events and TEAS-related adverse events shall be tabulated separately if present.

8. General Statistical Principles

- All statistical tests shall be two-sided unless otherwise specified.
- A P value less than 0.05 shall be considered statistically significant for descriptive interpretation.
- Because this is an exploratory study, no adjustment for multiplicity shall be performed unless a later approved amendment specifies otherwise. P values from exploratory analyses shall be interpreted descriptively.
- Continuous variables shall be summarized as mean ± standard deviation for normally distributed variables and as median (P25, P75) for non-normally distributed variables.
- Categorical variables shall be summarized as frequency and percentage, n (%).
- Percentages shall be calculated using the applicable analysis denominator and rounded to two decimal places unless otherwise specified.
- All statistical outputs shall be traceable to the locked analysis dataset and statistical code or software output file.

9. Statistical Methods

9.1 Normality assessment. The Shapiro-Wilk test shall be used to assess normality of continuous paired variables when relevant. Distributional shape, sample size, and clinical interpretability shall also be considered.

9.2 Baseline characteristics. Baseline characteristics shall be summarized descriptively. Continuous baseline variables shall be reported as mean ± standard deviation and range when appropriate. Categorical baseline variables shall be reported as counts.

9.3 Primary frequency and VAS outcomes. For paired comparisons of nausea frequency, nausea VAS severity, vomiting frequency, and vomiting VAS severity between the observation and TEAS intervention periods, the paired-sample t test shall be used for normally distributed paired differences. The Wilcoxon signed-rank test shall be used for non-normally distributed paired differences.

9.4 Response-rate analyses. Response rates shall be summarized for each assessment day as n (%) in the observation period and TEAS intervention period. For paired binary response outcomes, exact McNemar tests shall be used to compare response rates between periods at each assessment day. The 2 x 2 paired table shall classify each participant as response/response, response/non-response, non-response/response, or non-response/non-response. The discordant counts shall be reported as non-response to response and response to non-response.

9.5 Response-category distribution. Complete response, partial response, and no response shall be summarized by period and assessment day. Complete response corresponds to Grade 0, partial response corresponds to Grade I, and no response corresponds to Grade II or Grade III.

9.6 Simon two-stage decision rule. The Simon two-stage decision rule shall be applied to the prespecified binary decision endpoint: nausea response on day 1 during the TEAS intervention period, defined as Grade 0 or Grade I. The null response rate is 15% and the expected response rate is 30%. The first stage includes 23 evaluable participants. If three or fewer participants respond, the study stops for futility. If at least four participants respond, the study proceeds to the second stage. After completion of the second stage, a total of 48 evaluable participants are required; if 11 or fewer participants respond, the intervention is considered insufficiently effective, and if more than 11 participants respond, the efficacy criterion is met.

9.7 Directional grade-change analyses. Paired changes in symptom grades shall be summarized as improved, unchanged, or worsened for each assessment day. An overall patient-day summary may also be reported using 240 paired patient-days per outcome, derived from 48 participants across 5 assessment days. These analyses are exploratory and descriptive, and patient-days are not treated as independent observations for inferential testing unless a prespecified repeated-measures model is used.

9.8 Secondary outcomes. SF-36, SAS, and SDS scores at day 5 shall be compared between the observation and TEAS intervention periods using the paired-sample t test if paired differences are normally distributed or the Wilcoxon signed-rank test if not normally distributed. Results shall be reported as mean ± standard deviation or median (P25, P75), with corresponding test statistics and P values.

9.9 Safety analyses. TEAS-related adverse events and serious adverse events shall be summarized descriptively as counts, percentages, event type, timing, severity, relationship to TEAS, action taken, and outcome. No formal hypothesis testing is planned for safety outcomes.

10. Handling of Missing Data, Outliers, and Protocol Deviations

10.1 Missing data. No imputation is planned for missing primary or secondary outcomes unless required by an approved protocol amendment. The number of missing observations shall be documented for each endpoint and assessment day. Paired analyses shall include only participants with both observation-period and TEAS-intervention-period values for the relevant endpoint and day.

10.2 Outliers. Extreme values shall not be excluded solely because they are extreme. Values outside plausible clinical or instrument ranges shall be verified against source documents. Any correction or exclusion shall be documented in the analysis deviation log.

10.3 Protocol deviations. Major protocol deviations that may affect efficacy interpretation, such as changes in chemotherapy regimen, changes in antiemetic regimen, ineligible enrollment, or missing key paired assessments, shall be documented before final analysis and summarized in the study records.

10.4 Dropouts. Dropouts shall be handled according to the approved protocol. Excluded cases shall not be included in the final statistical analysis unless otherwise specified in a documented amendment.

11. Statistical Software and Reproducibility

Statistical analyses shall be performed using validated statistical software. The final submission record shall specify the software name and version: SPSS version 22.0.

All syntax, scripts, raw outputs, derived datasets, and final tables shall be archived in a study-specific analysis folder. File names shall include the study acronym, analysis type, version number, and date. Any manual calculation must be independently verified and documented.

12. Quality Control Procedure

1. Confirm that the analysis dataset matches the locked database and that all participant identifiers are de-identified in analysis outputs.

2. Verify inclusion in the first-stage, full efficacy, and safety analysis sets.
3. Check all grade-to-response derivations: Grade 0/I = response and Grade II/III = non-response.
4. Recalculate all percentages using the correct denominators: 23 for first-stage analyses, 48 for full-cohort daily analyses, and 240 patient-days for pooled 5-day directional summaries.
5. Verify all exact McNemar test inputs against paired 2 x 2 tables and confirm discordant counts.
6. Verify Wilcoxon signed-rank test outputs for frequency, VAS, SF-36, SAS, and SDS outcomes against the statistical output files.
7. Check consistency between statistical output, manuscript text, tables, abstract, and conclusions.
8. Document all changes after quality-control review in the version history table.

13. Output Specifications

Tables shall use the terminology "observation period" and "TEAS intervention period" consistently. Response tables shall report responders as n (%), absolute count differences where applicable, percentage-point differences, discordant paired counts, and exact McNemar P values. Frequency and VAS tables shall report median (P25, P75), paired test statistic, and P value. Safety outputs shall include event type, number of affected participants, action taken, and outcome.

14. Data Confidentiality and Record Retention

All analysis datasets and outputs shall be stored in password-protected or institutionally approved secure storage. Participant identifiers shall be de-identified in analysis files used for manuscript preparation. Statistical records, including the locked dataset, statistical code, quality-control checklist, analysis deviation log, and signed SOP, shall be retained according to institutional and journal requirements.

15. Change Control and Deviations from SOP

Any change to the planned statistical methods after SOP approval shall be documented in a version-controlled amendment or deviation log. The documentation shall include the date, description of change, reason, affected endpoints, impact on results, and approval by the principal investigator or corresponding author.

16. Analysis Method Mapping Table

Endpoint / analysis	Data type	Summary measure	Statistical method
Baseline characteristics	Continuous / categorical	Mean ± SD (range); n	Descriptive statistics
Nausea/vomiting frequency by day	Paired non-normal continuous/count-like	Median (P25, P75)	Wilcoxon signed-rank test unless paired differences are normal
Nausea/vomiting VAS by day	Paired continuous/ordinal-like	Median (P25, P75)	Wilcoxon signed-rank test unless paired differences are normal
Binary response by day	Paired binary	n (%), discordant counts	Exact McNemar test
Response categories	Categorical/ordinal	Complete response, partial response, no response, n	Descriptive statistics
Simon two-stage decision endpoint	Binary	Number of responders	Prespecified Simon two-stage decision rule
Directional grade change	Paired ordinal change	Improved / unchanged / worsened, n (%)	Descriptive paired patient-day analysis
SF-36, SAS, SDS	Paired continuous/score	Median (P25, P75) or mean ± SD	Wilcoxon signed-rank test or paired t test
Safety outcomes	Categorical/event data	n (%), event descriptions	Descriptive statistics

17. Version History

Version	Date	Author	Description of change	Approval
1.0	2025-04-25	Huiting Liu	Initial SOP for statistical analysis prepared for editorial submission.	Junfan Fang

18. Approval Signatures

Role	Name	Signature	Date
Prepared by / Statistician	Huiting Liu	Huiting Liu	25.4.25
Reviewed by / Data manager or co-investigator	Huiting Liu and Junfan Fang	Huiting Liu Junfan Fang	25.4.26
Approved by / Principal investigator or corresponding author	Junfan Fang	Junfan Fang	25.4.26
